# Peculiar Phosphonate Modifications of Velvet Worm Slime Revealed by Advanced Nuclear Magnetic Resonance and Mass Spectrometry

**DOI:** 10.1021/jacs.3c06798

**Published:** 2023-09-18

**Authors:** Alexandre Poulhazan, Alexander Baer, Gagan Daliaho, Frederic Mentink-Vigier, Alexandre A. Arnold, Darren C. Browne, Lars Hering, Stephanie Archer-Hartmann, Lauren E. Pepi, Parastoo Azadi, Stephan Schmidt, Georg Mayer, Isabelle Marcotte, Matthew J. Harrington

**Affiliations:** ‡ Department of Chemistry, Université du Québec à Montréal, Montreal, Quebec H2X 2J6, Canada; § Department of Zoology, Institute of Biology, University of Kassel, Kassel D-34132, Germany; ⊥ Department of Chemistry, McGill University, Montreal, Quebec H3A 0B8, Canada; || National High Magnetic Field Laboratory, Tallahassee, Florida 32310, United States; # Department of Biological and Chemical Sciences, University of the West Indies, Cave Hill Campus, Barbados BB11000, West Indies; ○ Complex Carbohydrate Research Center, University of Georgia, Athens, Georgia 30602, United States; △ Chemistry Department, Heinrich-Heine-Universität Düsseldorf, Düsseldorf D-40225, Germany

## Abstract

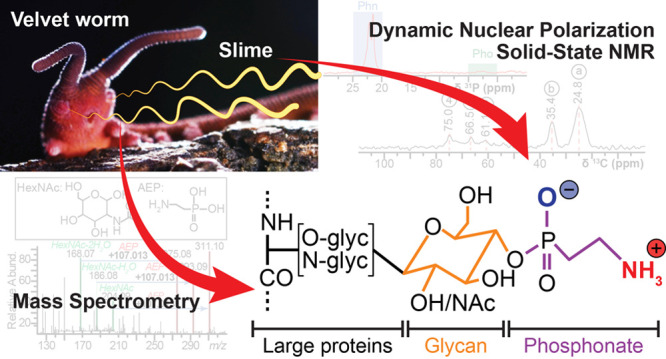

Nature is rich with examples of highly specialized biological materials produced by organisms for functions, including defense, hunting, and protection. Along these lines, velvet worms (Onychophora) expel a protein-based slime used for hunting and defense that upon shearing and dehydration forms fibers as stiff as thermoplastics. These fibers can dissolve back into their precursor proteins in water, after which they can be drawn into new fibers, providing biological inspiration to design recyclable materials. Elevated phosphorus content in velvet worm slime was previously observed and putatively ascribed to protein phosphorylation. Here, we show instead that phosphorus is primarily present as phosphonate moieties in the slime of distantly related velvet worm species. Using high-resolution nuclear magnetic resonance (NMR), natural abundance dynamic nuclear polarization (DNP), and mass spectrometry (MS), we demonstrate that 2-aminoethyl phosphonate (2-AEP) is associated with glycans linked to large slime proteins, while transcriptomic analyses confirm the expression of 2-AEP synthesizing enzymes in slime glands. The evolutionary conservation of this rare protein modification suggests an essential functional role of phosphonates in velvet worm slime and should stimulate further study of the function of this unusual chemical modification in nature.

Velvet worms comprise an ancient group of terrestrial invertebrates, including about 230 described species. The two major subgroups, Peripatidae and Peripatopsidae, diverged about 380 MYA.^1^ Velvet worms capture their prey by projecting sticky slime from the papillae on each side of their head^2^ (Movie S1). This gel-like slime, primarily comprised of proteins, transforms into solid fibers under mechanical shearing and rapid drying. The struggling of the ensnared prey accelerates hardening into glassy fibers with a stiffness comparable to Nylon.^3^ These biopolymeric fibers can be solubilized in water, and new indistinguishable fibers can be drawn mechanically *in vitro* from the resulting solution.^3^ The mechanism for reversible fiber formation is thus encoded in the chemical structure of the proteins. Indeed, mechanoresponsive fiber formation outside the animal’s body under ambient conditions and their recyclability provides a promising avenue for bioinspired development of sustainable plastics and glues.^3^ Yet, many questions remain regarding slime composition and underlying biochemical mechanisms guiding reversible fiber formation.

Previous biochemical analyses from several onychophoran species have revealed a primarily proteinaceous composition with components of different sizes.^4−6^ Mid-molecular-weight (MMW) proteins and small quantities of lipids (<1%) were proposed to form condensed nanodroplets,^4^ while low-molecular-weight (LMW) proteins are proposed to act as antimicrobial components.^6^ However, several high-molecular-weight (HMW) proteins were shown to be the major structural component of slime fibers.^3−8^ Based on positive phosphostaining and elemental analysis of the HMW proteins from the Peripatopsidae species *Euperipatoides rowelli*, as well as the high content of divalent cations (Mg^2+^, Ca^2+^), phosphate-mediated electrostatic interactions were hypothesized to drive reversible fiber formation.^7,9^ However, the prediction of phosphorylated amino acids in *Eu. rowelli* slime was solely based on bioinformatics analyses, and never experimentally confirmed.^7^ Moreover, similar analysis of HMW slime protein sequences from a Peripatidae species collected in Singapore did not detect phosphorylation sites.^10^

Here, we elucidated the chemical nature of the slime’s phosphorus content in two distantly related velvet worm species using natural abundance NMR spectroscopy and heteronuclear dynamic nuclear polarization (DNP) experiments with magic-angle spinning (MAS), in combination with higher-energy collision-induced dissociation (HCD) tandem mass spectrometry (MS/MS) analysis of glycan protein modifications. We demonstrate that in both species—the peripatopsid *Eu. rowelli* and the peripatid *Epiperipatus barbadensis*—large slime proteins possess an extremely rare post-translational modification consisting of phosphonated glycans. The occurrence of this protein modification in both species indicates a highly conserved feature over at least 380 MY, suggesting a critical functional role in the slime storage, fiber formation, and/or adhesion.

Phosphorus is ubiquitous in living organisms and typically found as phosphate esters (C–O–P bond),^11^ and less frequently as phosphonates (C–P bond) in natural organophosphorus compounds.^12−14^ We applied solution and solid-state (ss) ^31^P NMR experiments to differentiate between these forms in the slime from *Eu. rowelli* (Figures 1a, b) and *Ep. barbadensis* (Figures 1c, d).^1,13^ Several intense ^31^P NMR signals appear at 20–23 ppm (Figures 1b and d), which are unambiguously assigned to phosphonates and are not environmental contaminations (Figure S1). Additional weaker phosphate peaks between 0–5 ppm, only found in *Eu. rowelli*, are ascribed to phosphoproteins rather than phospholipid phosphate esters, considering the low lipid abundance (Figure S2).^7^ Quantitative peak analysis reveals that *Eu. rowelli*‘s slime contains 17 times more phosphonates than phosphates (Figure 1b), while phosphates are essentially absent in *Ep. barbadensis* (Figure 1d). The ^31^P NMR spectra reveal a difference in the phosphonate region, with three peaks being detected in *Eu. rowelli*’s slime at 22.2/21.6/21.0 ppm while the 21.0 ppm peak is absent in *Ep. barbadensis*. This suggests subtle differences between their phosphonate environments and may indicate evolutionary variations between the two onychophoran subgroups. In addition, the lack of phosphates in the slime of *Ep. barbadensis* is consistent with the lack of phosphorylation sites detected in the HMW proteins of the Singapore velvet worm (*Eoperipatus* sp., a representative of Peripatidae).^10^

**Figure 1:**
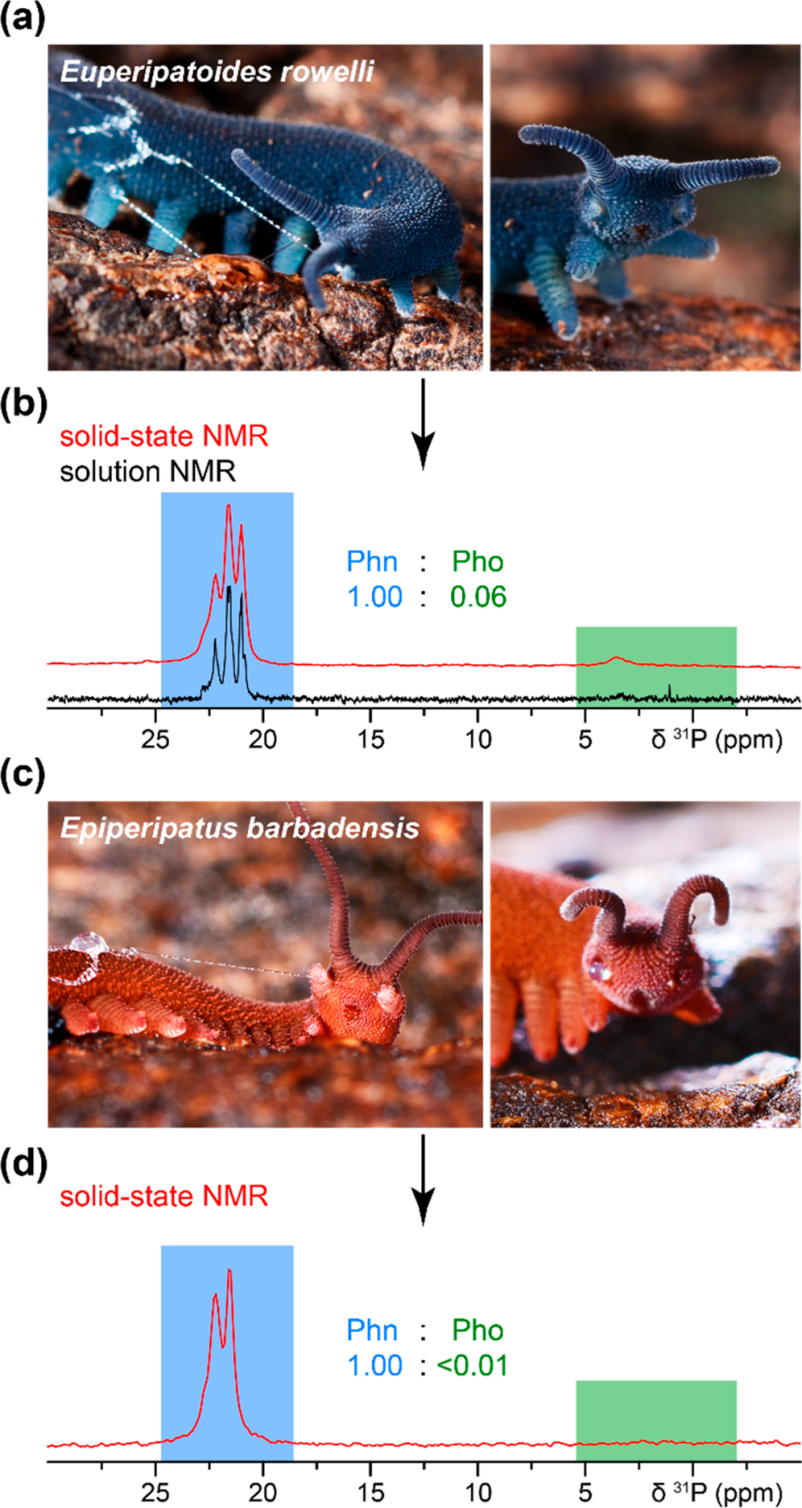
^31^P ssNMR reveals phosphonates in the slime of two distantly related onychophoran species. Photographs of (a) the peripatopsid *Euperipatoides rowelli* and (b) corresponding solution (black) and solid-state (red) ^31^P NMR spectra indicate predominant phosphonates (Phn, highlighted in blue) compared to phosphate ester (Pho, green). (c) Photographs of the peripatid *Epiperipatus barbadensis* and (d) corresponding slime ^31^P ssNMR spectrum shows only phosphonates.

These results provide strong evidence that the high phosphorus content in velvet worm slime previously ascribed to phosphorylated proteins^7,9^ is, rather, associated with phosphonate-rich molecules. These findings apply to both fibrilized and nonfibrilized slime, as revealed for *Eu. rowelli* (Figure 1b and Table S1). Phosphonates were also detected in the bodies of both species by ssNMR (Figure S3). The occurrence of natural phosphonates is well supported for various marine and freshwater organisms; whereas phosphonate-containing moieties have only rarely been detected in terrestrial invertebrates (see literature review of natural phosphonates in Table S2). We thus proceeded to a detailed characterization of the phosphonate moiety and its association with slime proteins.

Comparison of 1D ^1^H (^31^P-decoupled) and ^1^H–^31^P TOCSY (total correlation spectroscopy) solution NMR spectra of *Eu. rowelli* slime to those of several phosphonate standards^15^ revealed that they are in good agreement with 2-aminoethylphosphonate (2-AEP) (Figures S4 and S5, and Table S1). In marine microorganisms, the biosynthesis pathway of 2-AEP is catalyzed by phosphoenolpyruvate mutase (PEPm), phosphoenolpyruvate decarboxylase (Ppd) and 2-aminoethyl phosphonate transaminase (AEPt).^16−18^ Local BLAST searches of published protein sequences of these three enzymes^17^ against transcriptomes of *Eu. rowelli* and *Principapillatus hitoyensis* (representative of Peripatidae, like *Ep. barbadensis*) revealed that PEPm-, Ppd- and AEPt-encoding genes are expressed in the slime glands of both onychophoran species (Tables S3–S6), supporting the ability of velvet worms to produce 2-AEP phosphonate moieties. In addition to the slime gland, these genes are expressed in several other tissues, consistent with the detection of phosphonates in various parts of the body of the worm (Table S4 and Figure S3), suggesting a role of 2-AEP in other biological functions.

^1^H and ^31^P solution NMR diffusion experiments on *Eu. rowelli* slime revealed that phosphonates are associated with large molecules (Figure S6). On the other hand, lipid extraction, ^13^C ssNMR, and phenol-sulfuric acid assay show low amounts of lipids and glycans in the slime^7^ (Figures S1 and S7), excluding phosphonate modification of lipids or pure polysaccharides. Therefore, phosphonates are most likely associated with HMW proteins. ^31^P ssNMR experiments on HMW (>300 kDa), MMW (100–300 kDa), and LMW (8–100 kDa) fractions obtained from a triple dialysis of the slime confirm this hypothesis (Figure 2). Phosphonates are indeed associated with molecules above 100 kDa, while LMW compounds contain phosphates (Figure 2b). According to previous SDS-PAGE analyses, this includes HMW monomers/complexes (232–429/478–634 kDa) or MMW (110 kDa) proteins in *Eu. rowelli* slime.^4,10^

**Figure 2:**
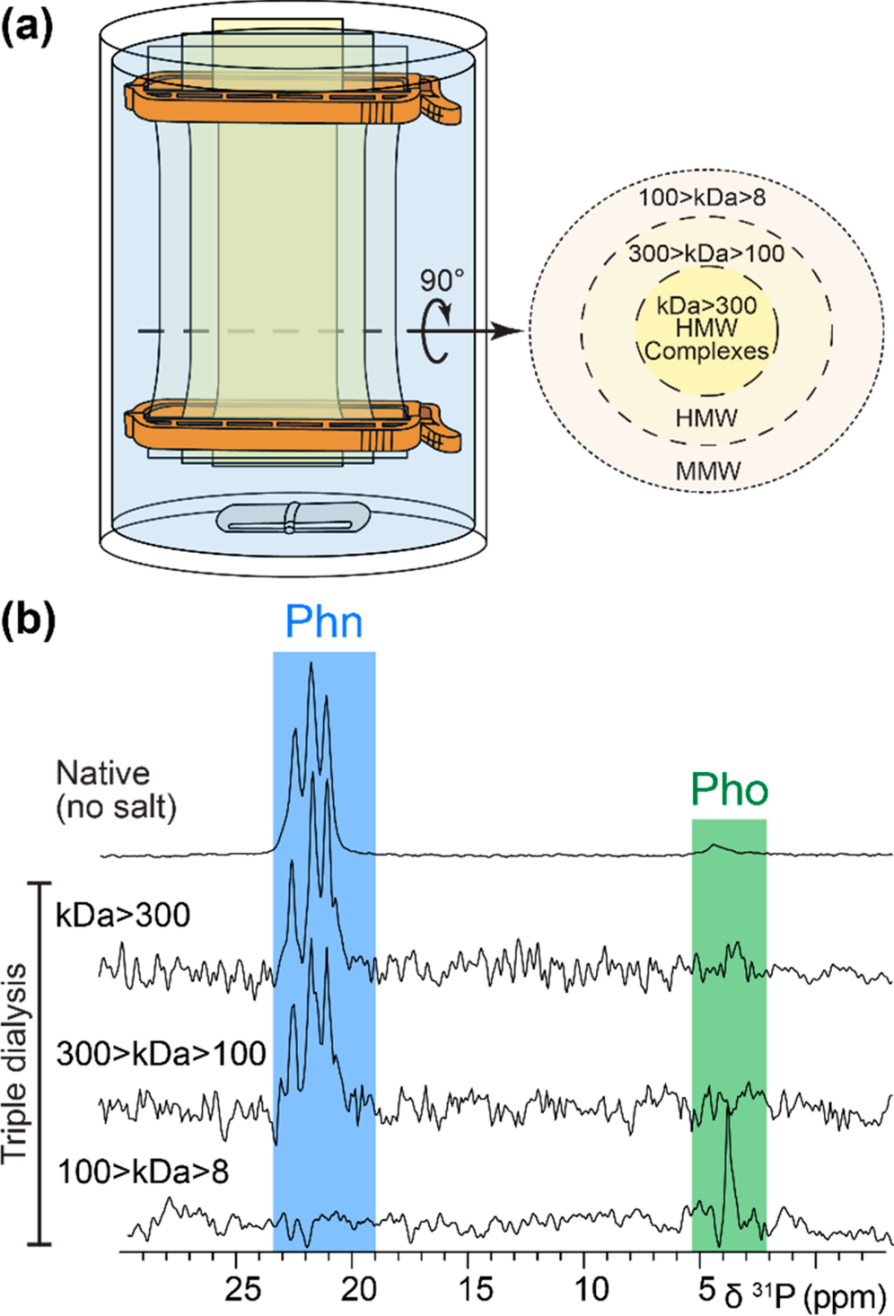
Triple dialysis of *Eu. rowelli* slime followed by ^31^P ssNMR. (a) Simultaneous triple dialysis setup. (b) Dialysis fractions analyzed by ^31^P ssNMR show that HMW proteins contain most of the phosphonates (Phn), while LMW proteins (<100 kDa) lack phosphonates but contain phosphates (Pho).

Deeper structural analysis of the 2-AEP moieties was performed by MAS-DNP and HCD-MS/MS. MAS-DNP provides enhanced sensitivity (Figure 3a and Figure S8), enabling detection and identification of carbon signals in endogenous abundance, and determination of proximity between carbon and phosphorus atoms by monitoring the magnetization transfer from ^31^P to ^13^C during cross-polarization (CP); as duration increases, carbons further from the phosphorus atom gradually appear on the spectra (Figure 3a, b). The 2D ^31^P–^13^C CP MAS-DNP ssNMR experiments performed on slime further confirm that ^13^C–^31^P contacts arise from phosphonates rather than from phosphorylation (Figure S9). Furthermore, carbons closer to the phosphonate moieties have chemical shifts of 24.8 and 35.4 ppm (Figure 3b and Figure S10), which agrees well with 2-AEP’s structure (Table S1). This is further confirmed by the natural abundance ^15^N MAS-DNP ssNMR spectrum in which the signal at ∼31 ppm could correspond to 2-AEP’s amine (Figure S11). Carbons further from the phosphorus atom appear at 63.3/75.0 ppm in *Eu. rowelli*, and 61.1/66.5/75.0 ppm in *Ep. barbadensis*, which are typical of glycans (Figure 3a, b, Table S7),^16,19^ indicating the presence of phosphonated glycans in the slime. Additionally, an intermolecular contact with a carbon at 44.4 ppm, possibly corresponding to arginine carbon side chain (Figure 3a, Figures S10–S12) was detected only in *Eu. rowelli*’s slime. DFT calculations (Figure S13) support an ∼2.9 Å distance between 2-AEP’s phosphorus and arginine.

**Figure 3:**
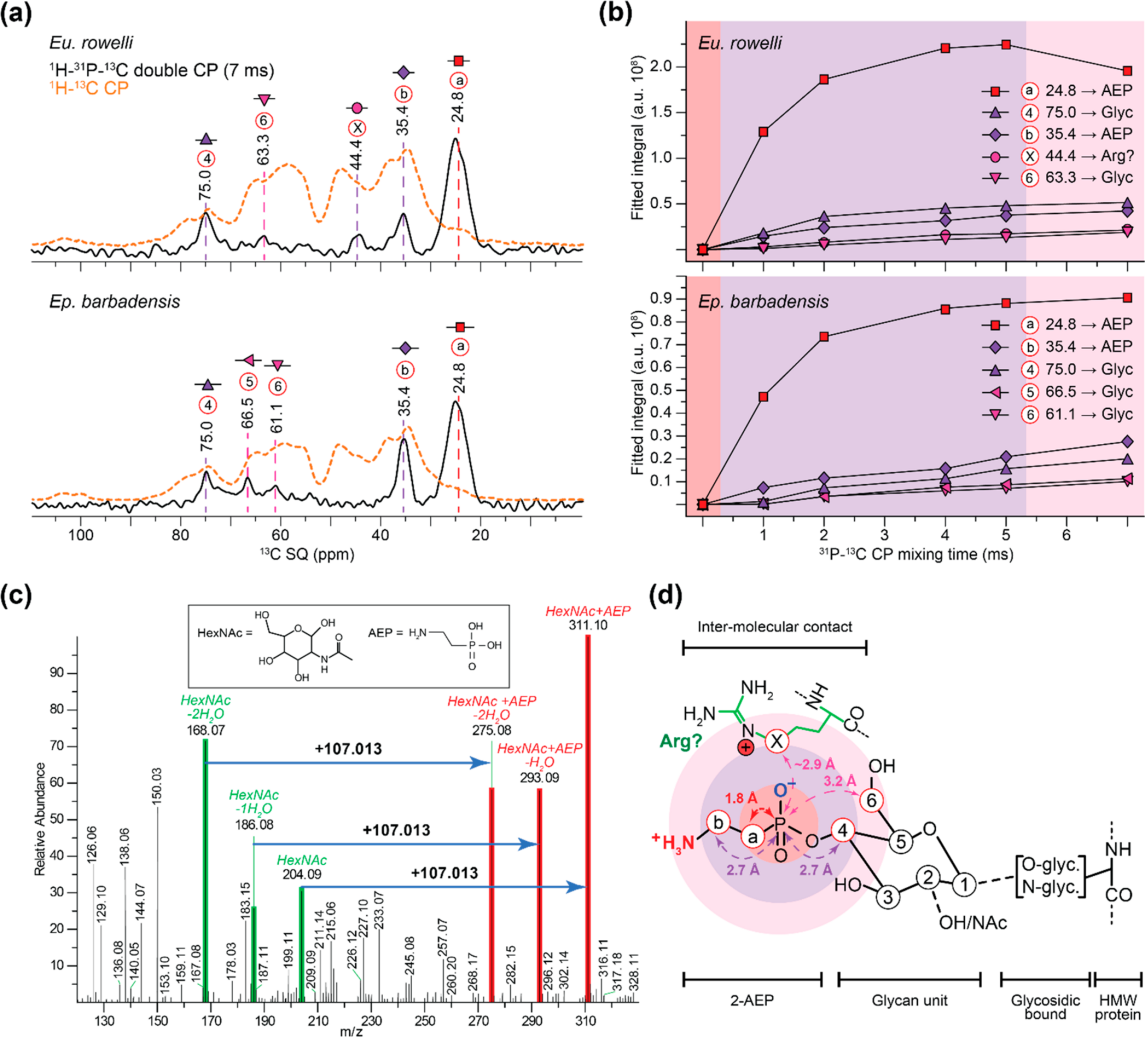
MAS-DNP and HCD-MS/MS identify phosphonoglycans in velvet worm slime. (a) MAS-DNP ^1^H–^31^P–^13^C double CP spectra reveal carbon atoms close to ^31^P atoms (black line) as compared to direct ^1^H–^13^C CP spectra (orange dashed line) showing all carbons. (b) ^13^C peak intensities as a function of CP mixing time shows the sequential proximity of the ^31^P atom to ^13^C. (c) HCD-MS/MS fragmentation of tryptic peptides in *Eu. rowelli* are indicative of 2-AEP modified HexNAc glycans attached to slime proteins. (d) Schematic representation of phosphonoglycans decorating slime proteins. Distances were estimated using MAS-DNP CP build-ups of *Eu. rowelli* and density-functional theory; *X* atom represents possible intermolecular contact with arginine.

The 2-AEP modification of glycans associated with slime proteins is further corroborated by HCD-MS/MS analyses of trypsin-digested *Eu. rowelli* slime. The results show oxonium ions revealing both unmodified and 2-AEP-modified N-acetylhexosamine (HexNAc) decorating tryptic peptides (Figures 3c and Figure S14). Previous biochemical analyses of the peripatopsid *Eu. kanangrensis* assumed that carbohydrates mostly occur as N-acetyl galactosamine (GalNAc)^5^ bound to slime proteins via O-glycosylation. However, the exact linking pattern and nature of the carbohydrate units require further investigation (Figure 3d).

The physicochemical properties of glycans and, by extension, the possible interactions with charged amino acids will be altered by 2-AEP functionalization.^20,21^ At the native pH of 5.2 for the ejected slime,^3^ the phosphonate moiety is most likely in a zwitterionic charge state (Figure S15), consistent with previous work highlighting the role of electrostatic interactions between slime proteins during storage and fiber formation.^7^ The local charge density in the HMW proteins should increase their solubility, while also enabling electrostatic interactions with divalent ions present at elevated concentrations in the slime.^4^ This chemical strategy resembles that observed in well-studied biological adhesives derived from mussels and sandcastle worms. These materials are enriched in charged amino acid residues, as well as post-translational protein modifications such as 3,4-dihydroxyphenylalanine (DOPA) and phosphoserine, which are crucial for material formation and function.^22,23^ Electrostatic interactions are especially important in these systems for influencing phase separation of proteins, which functions in storage, transport, and eventual solidification into functional glues.^22−24^ The charged phosphonate moieties discovered here may thus contribute to onychophoran slime storage and its transition to recyclable biopolymeric fibers.

The occurrence of phosphonates in slimes from distinct onychophoran subgroups suggests that phosphonate production has been evolutionarily conserved for at least 380 MY and might be shared by all existing onychophoran species. Given the large metabolic cost to produce this modification,^16^ the evolutionary conservation of phosphonate production suggests an important role in the formation and function of slime fibers. More generally, natural phosphonates have been reported across various taxonomic groups of organisms, associated with small organic molecules, glycans, lipids, or decorating biomolecules such as glycolipids, glycoceramides, and glycoproteins with diverse functions (see detailed information and references in Table S2). Given that phosphonate producers have been predominantly reported in aquatic environments, our findings suggest that the prevalence of natural phosphonates in terrestrial organisms may be underestimated.^14,16,17,25^ Notably, the ancestors of velvet worms (and their closest extant relatives, water bears and arthropods) were extinct lobopodians that mostly, if not exclusively lived in marine habitats.^26^ Therefore, phosphonate production might be an ancestral feature inherited from marine lobopodians, and it seems probable that phosphonates may be detected in other descendants of this lineage, including tardigrades and various arthropods (as already confirmed for migratory locusts; Table S2).

In conclusion, this work describes a rare, presumably charged phosphonoglycan modification (containing 2-AEP) of HMW proteins in onychophoran slime. Verifying the potential functions of this modification requires further investigation, but our key insights into the molecular composition and assembly of velvet worm slime may help to inspire the design of sustainable polymers and adhesives. Furthermore, the discovery of phosphonates in another terrestrial invertebrate underlines the necessity to consider phosphonates as a potential source of organophosphorus in understudied groups of animals and their biological functions.

## Supporting Information Available

Materials and Methods; control for bacteria and environment contamination of the slime samples (Figure S1); Mobility selective 13C NMR spectra of *Eu. rowelli* native aggregated slime fibers (Figure S2); ^31^P NMR to detect phosphonates in slime and tissues of both velvet worm species (Figure S3); frequency-specific ^31^P decoupled ^1^H solution NMR spectra (Figure S4); 2D ^1^H–^31^P hetero TOCSY for coupling in phosphonate standards and slime (Figure S5); ^1^H and ^31^P DOSY of *Eu. rowelli* slime locate phosphonates in large objects (Figure S6); lipid extraction from slime demonstrates phosphonates in protein fraction (Figure S7); Sensitivity of NMR experiments and MAS-DNP enhancement methodology (Figure S8); 2D ^13^C–^31^P contacts as detected by 2D MAS-DNP on native slime in *Eu. rowelli* (Figure S9); comparison of MAS-DNP ^31^P–^13^C build-up spectra of slimes from both species (Figure S10); MAS-DNP natural abundance ^15^N for protein and 2-AEP identification (Figure S11); ^13^C–^13^C MAS-DNP double quantum/single quantum correlations in the slime (Figure S12); DFT calculated 3D structure representation of the phosphonate in the slime (Figure S13); HCD-MS/MS chromatogram of peptide with 2-AEP-glycan modification (Figure S14); 2-AEP charge and corresponding phosphonates in velvet worm slime (Figure S15); ^31^P, ^13^C and ^1^H chemical shift of phosphonate standards and slime (Table S1); known natural phosphonate moieties (Table S2); sequencing and assembly statistics of tissue-specific transcriptomes from *Eu. rowelli* and *P. hitoyensis*. (Table S3); local BLAST of phosphonate biocatalytic enzymes in the spirochete bacterium *Treponema denticola* (Table S4); best matches of protein BLAST of phosphonate enzymes in velvet worms (Table S5); deduced protein sequences of cloned phosphonate enzyme genes from *Eu. rowelli* and *P. hitoyensis* (Table S6); comparison of slime and reported glycophosphonate chemical shifts (Table S7) (PDF)
Movie S1, slow motion video of slime ejection by a living specimen of *Ep. barbadensis* (AVI)

## Supplementary Material

ja3c06798_si_001.pdf

ja3c06798_si_002.avi

## Abbreviations

NMR: nuclear magnetic resonance
2-AEP: 2-aminoethyl phosphonate
MMW: mid-molecular-weight proteins
LMW: low-molecular-weight proteins
HMW: high-molecular-weight proteins
MS: mass-spectrometry
Phn: phosphonate
Pho: phosphate
ssNMR: solid-state NMR
PEPm: phosphoenolpyruvate mutase
Ppd: phosphoenolpyruvate decarboxylase
AEPt: 2-aminoethyl phosphonate transaminase
MAS-DNP: magic-angle spinning combined with dynamic nuclear polarization
HCD MS/MS: higher-energy collision induced dissociation combined with tandem mass spectrometry
CP: cross-polarization
HexNAc: N-acetylated hexose
GalNAc: N-acetyl galactosamine
DOPA: 3,4-dihydroxyphenyalanine
MAS: magic-angle spinning
DFT: density-functional theory
CID: collisional-induced dissociation
