# Supplementary material for: Peculiar Phosphonate Modifications of Velvet Worm Slime Revealed by Advanced Nuclear Magnetic Resonance and Mass Spectrometry
Source: J Am Chem Soc. 2023 Sep 18;145(38):20749–54. doi: 10.1021/jacs.3c06798 (PMC10540779; doi:10.1021/jacs.3c06798)
Supplement: Supplementary file 1 — ja3c06798_si_001.pdf [file ja3c06798_si_001.pdf]

Supporting Information

Peculiar Phosphonate Modifications of Velvet Worm Slime Revealed by Advanced Nuclear Magnetic Resonance and Mass Spectrometry

Alexandre Poulhazan[†,‡], Alexander Baer[⊥,‡], Gagan Daliaho[§], Frederic Mentink-Vigier[||], Alexandre A. Arnold[†], Darren C. Browne[#], Lars Hering[⊥], Stephanie Archer-Hartmann[⊥⊥], Lauren E. Pepi[⊥⊥], Parastoo Azadi[⊥⊥], Stephan Schmidt[§§], Georg Mayer[⊥], Isabelle Marcotte[†,*], Matthew J. Harrington[§,*].

† Department of Chemistry, Université du Québec à Montréal, Montreal, Quebec H2X 2J6, Canada.

⊥ Department of Zoology, Institute of Biology, University of Kassel, D-34132, Germany.

§ Department of Chemistry, McGill University, Montreal, Quebec H3A 0B8, Canada.

|| National High Magnetic Field Laboratory, Tallahassee, Florida 32310, United States.

Department of Biological and Chemical Sciences, University of the West Indies, Cave Hill Campus, Barbados, BB11000, West Indies.

⊥⊥ Complex Carbohydrate Research Center, University of Georgia, Athens, Georgia 30602, United States.

§§ Chemistry Department, Heinrich-Heine-Universität Düsseldorf, D-40225, Germany.

‡ These authors contributed equally to this work

* Corresponding authors: marcotte.isabelle@uqam.ca; matt.harrington@mcgill.ca

Table of Contents

Material 3
Collection of specimens 3
Export permits 3
Methods 3
Collection and rehydration of samples 3
Preparation and acquisition of samples for NMR 3
MAS-DNP acquisition 4
Density Functional Theory (DFT) calculations 4
Transcriptomic analyses 5
Cloning of phosphonate protein genes from cDNA 5
Trypsin digestion of slime samples 6
Bligh and Dyer lipid extraction 6
Additional text highlighting differences between two species of velvet worms 6
Figure S1. Control for bacteria and environment contamination of the slime samples 7
Figure S2. Mobility selective ^{13}C NMR spectra of *Eu. rowelli* aggregated slime fibers 8
Figure S3. ^{31}P NMR to detect phosphonates in slime and tissues of both species 9
Figure S4. Frequency-specific ^{31}P decoupled ^{1}H solution NMR spectra 10
Figure S5. 2D ^{1}H-^{31}P hetero TOCSY for coupling in phosphonate standards and slime. 11
Figure S6. ^{1}H and ^{31}P DOSY of *Eu. rowelli* slime locate phosphonates in large objects. 12
Figure S7. Lipid extraction from slime demonstrates phosphonates in protein fraction.. 13
Figure S8. Sensitivity of NMR experiments and MAS-DNP enhancement. 14
Figure S9. ^{13}C-^{31}P contacts as detected by 2D MAS-DNP on slime in *Eu. rowelli*. 15
Figure S10. Comparison of MAS-DNP ^{31}P-^{13}C build-up spectra of both slimes 16
Figure S11. MAS-DNP natural abundance ^{15}N for protein and 2-AEP identification. 17
Figure S12. Slime ^{13}C-^{13}C MAS-DNP Double Quantum/Single Quantum correlations.. 18
Figure S13. DFT calculated 3D structure representation of the phosphonate in slime 19
Figure S14. HCD-MS/MS chromatogram of peptide with 2-AEP-glycan modification.. 20
Figure S15. 2-AEP charge and corresponding phosphonates in velvet worm slime. 21
Table S1. ^{31}P, ^{13}C and ^{1}H chemical shift of phosphonate standards and slime 22
Table S2. Known natural phosphonate moieties 23
Table S3. Sequencing and assembly statistics of tissue-specific transcriptomes 26
Table S4. Local BLAST of phosphonate biocatalytic enzymes in the spirochete bacterium *Treponema denticola* 27
Table S5. Best matches of protein BLAST of phosphonate enzymes in velvet worms ... 28
Table S6. Deduced protein sequences of cloned phosphonate enzyme genes from *Eu. rowelli* and *P. hitoyensis*. 29
Table S7. Comparison of slime and reported glycophosphonate chemical shifts 30
Movie S1. Slow motion video of slime ejection by a specimen of *Ep. barbadensis* 31
References 32

Material

All chemicals, including enzymes, chemicals, phosphate and phosphonate standards, were purchased from Sigma-Aldrich Co. (Oakville, ON, Canada). They were used without further purification. Dialysis tubings (300 kDa, 100 kDa and 3 kDa cutoffs) were obtained from VWR international (Mississauga, ON, Canada) and rinsed in Nanopure® water prior to use.

Collection of specimens

Specimens of *Euperipatoides rowelli* Reid, 1996 were collected in Tallaganda State Forest, 35°26'S, 149°33'E, New South Wales, Australia. Specimens of *Epiperipatus barbadensis* (Froehlich, 1962) were collected in Hope Road Gully 13°08'51"N, 59°33'12"W, St. George, Barbados. Specimens of *Principapillatus hitoyensis* Oliveira *et al.*, 2012 were collected in Reserva Biológica Hitoy Cerere, 09°40'21"N, 83°02'36"W, Province of Limón, region of Talamanca, Costa Rica

Export permits

Specimens were collected and exported under the following permits: (i) SL101720/2016, issued by NSW National Parks and Wildlife Service, Australia, and PWS2016-AU-001023 and Department of Sustainability, Environment, Water, Population and Communities, Australia, (ii) 8434/56/1, Ministry of Environment and National Beautification, Green and Blue Economy, Barbados and (iii) 123-2005-SINAC and 014950, Gerencia Manejo y Uso Sostenible de RR NN–Ministerio del Ambiente y Energia, Costa Rica. All animal treatments complied with the principles of laboratory animal care and the local laws on the protection of animals.

Methods

Collection and rehydration of slime samples

Slime of *Eu. rowelli* was harvested in Germany and shipped to Canada for chemical treatment, dialysis and NMR analysis using express courier. Native slime was preferred for shipping, but dry samples proved to provide similar results for both solid-state and solution NMR using 35 °C bath followed by circular rotation using Glas-Col (Terre-Haute, IN, USA) hardware to rehydrate dry slime. Slime of *Ep. barbadensis* was harvested and analyzed in Canada without long-distance transportation. All NMR analyses on native slime were performed on at least three batches of slime for biological consistency.

Preparation and acquisition of samples for NMR

Liquid slime samples were successfully filled into an NMR tube (diameter 5 mm) without forming fibers. This was performed first by diluting the native slime in D_2O increasing the fluidity and preventing partial aggregate formation. The slime appeared to aggregate during insertion into standard 7 inches-long NMR tubes. The aggregation could be considerably reduced by shortening the NMR tubes to ca. 4 inches. To form aggregates amenable to solid state NMR (ssNMR), the slime was simply agitated and mixed using a spatula. Aggregated slime appeared as a white sticky, gum-like material which was recovered and packed into an ssNMR rotor. ^{31}P NMR spectra of solid samples were recorded using a single 4 µs 90° pulse followed by spinal-64 ^{1}H decoupling at a radio-frequency field of 38 kHz, up to 30,000 scans were recorded with a recycle delay of 3 s. ^{31}P NMR spectra of soluble samples were recorded using a single 15 µs 90° pulse followed by waltz-16 decoupling at a ^{1}H radio-frequency field of 3.5 kHz, 400 scans were recorded with a recycle delay of 5 s. The ^{31}P-^{1}H heteronuclear TOCSY were recorded using a 70 ms-long DIPSI2 spin lock. The spectral widths were 50 and 10 ppm for ^{31}P and ^{1}H, respectively. Spectra of the phosphonate standards were recorded with 16 scans while the slime samples required 128, in both cases the indirect dimension consisted of 128 increments. The NMR pulse

sequences used in this study are detailed in previously published studies[1-3]. All ssNMR ^{13}C chemical shifts (including those acquired by MAS-DNP) were obtained using 15 μs and 3 μs for solution and ssNMR, respectively, and spectra were externally referenced to adamantane's CH_2 downfield signal set to 38.48 ppm[4], while ^{31}P chemical shifts were referenced using an 85% aqueous solution of phosphoric acid (H_3PO_4) set to 0 ppm[5], and ^{15}N chemical shifts were referenced using an NH_4Cl powder set to 24.9 ppm[6].

MAS-DNP acquisition

For MAS-DNP enhanced experiments, samples were prepared similarly to ssNMR, except that aggregates were mixed with a radical that contained unpaired electrons. The sample also contained a cryoprotectant to avoid the formation of ice crystals and preserve potentially fragile structures. A radical stock solution of AMUPol or AsymPol-POK[7-10] was respectively used for *Eu. rowelli* and *Ep. barbadensis*, freshly prepared in d_8-glycerol/D_2O/H_2O (60/30/10 vol%), referred to as the *DNP juice* or the DNP matrix, at a final radical concentration of 10 mM. To prepare the MAS-DNP slime sample, 50 μL of the stock solution were added to ~50 μL of the slime and mixed for 5–10 min to allow the radical solution to penetrate the sample. Around 35 mg of well-hydrated samples were transferred into a 3.2-mm sapphire rotor. All MAS-DNP experiments were performed on a 600 MHz/395 GHz MAS-DNP spectrometer equipped with a gyrotron microwave source[11-12] using a 3.2 mm, custom-made, HXY probe at 8 kHz MAS frequency. The cathode currents of the gyrotron were 160 mA. The temperature was ~98 K with the microwave (MW) off and ~100 K with the micro-wave on. The radical in the *DNP juice* contained free electrons that had a 658-fold higher spin polarization than ^{1}H nucleus. Under the microwave radiation, the electron spin polarization was transferred to the nuclei and the sample remains at a cryogenic temperature. Typically, a 60-fold enhancement factor of NMR sensitivity with and without microwave irradiation was achieved (**Figure S8b**). Relatively short buildup time constants (1.4 s) indicated a sufficient mixing of the radicals and biomolecules in these cellular samples. 2D correlation experiments were implemented with 50-ms DARR, 1.5-s PDSD, NCa, and 100-ms NCaCx experiments. The total time for each 2D spectrum was 6 h, 8 h, and 2 h for the DARR, PDSD, and NC experiments respectively.

Density Functional Theory (DFT) calculations

DFT gave the optimized resulting geometry as follow: 43
Coordinates from ORCA-job r2SCAN-3c

C	-6.28617023264502	2.38139257254500	0.09243181387826
C	-6.23319597485045	2.05327301370780	-1.38556430750551
N	-7.27550585414025	2.87841114782770	-2.14580934073389
H	-6.45842569746577	1.00363945817936	-1.59586426540360
H	-5.25985091576539	2.30364389033574	-1.81777404582538
P	-4.85694149664255	1.58725438452218	0.92302550383774
H	-7.21390017197148	2.04681727584072	0.57229967294640
H	-6.17813314256961	3.45819189104783	0.28289351500408
O	-3.63485870684023	1.67799529632120	0.08139108375378
H	-8.22105262005466	2.67905642835347	-1.80843775303848
H	-7.25085178661586	2.68537651805075	-3.15081994348680
O	-5.45034616040685	0.12161862425147	1.19069124995645
O	-4.77945536760181	2.21003189301643	2.38541050875373
H	-6.76208634838106	5.41292958969770	2.60973760554928
O	-6.27137323736572	4.64150058819208	2.29104721501860
N	-5.55637861957412	6.59330711900119	4.30134812242205
H	-4.22278539502546	6.26693046614186	2.76805314277530
C	-5.22043373738608	4.41827973660760	3.23504736354171

C	-4.57274285981645	5.73429613465205	3.66302047498798
H	-2.58163442753997	7.20089721397166	4.19223277010100
H	-2.10132820374292	1.52660210370726	2.75477190350441
C	-3.34481020733187	5.46294034004159	4.54734859661472
O	-2.71403278248025	6.62107603527916	4.95654440348768
O	-2.43344848011701	4.66279410434161	3.79514101659690
C	-2.94048661914480	3.37177617888495	3.48282893620705
C	-1.82132251284830	2.58991397609621	2.79525907713400
H	-3.63189204407957	4.92452594300130	5.47047891361482
H	-0.93131320421292	2.67741006501889	3.42887374835569
H	-2.00077233047284	2.59996779948339	0.85640169214354
O	-1.50115456015412	3.10244480059304	1.51587011448705
C	-4.17419137744821	3.53402170162016	2.59249141512786
H	-5.62133990259181	3.89132171624697	4.12082274848909
H	-3.23203287104229	2.83649887141554	4.40737965995110
H	-3.85518810619690	3.96134067580849	1.63122233858001
H	-7.10612484482561	3.87941502559830	-2.01265106192430
H	-4.77845883448240	-0.54680182638191	1.39311357462068
C	-5.47734222755223	7.96940102499645	4.11921886104275
H	-5.91778084125749	6.28382552862959	5.19471192476024
C	-6.30095825198778	8.79179488514959	5.07753396247000
H	-6.69432489610850	9.66258116779924	4.55050201535245
H	-5.63891098885725	9.15620943847166	5.87120907124848
H	-7.12058375354267	8.23243785038924	5.53697924386092
O	-4.79491940686278	8.46103935154501	3.23712745774188

Transcriptomic analyses
Total RNA was extracted using TRIzol (Thermo Scientific, Waltham, MA, USA) and the RNeasy MinElute Cleanup Kit (Qiagen, Hilden, Germany) according to the manufacturers' protocols. Library preparation (purification of poly-A containing mRNA molecules from total RNA, mRNA fragmentation, random primed strand specific cDNA synthesis, adapter ligation and adapter specific PCR amplification) and sequencing (Illumina NovaSeq 6000 S4 PE150 XP, paired end, 2 x 150 bp) was performed by a sequencing company (Eurofins Genomics GmbH, Konstanz, Germany). Raw reads were quality filtered and trimmed using Trimmomatic v.0.39[13] (ILLUMINA CLIP:TruSeq3-PE-2.fa:2:30:10 LEADING:30 TRAILING:30 SLIDINGWINDOW:4:30 MINLEN:50). To validate the filtering step, the raw reads were quality checked before and after trimming using FastQC v.0.11.9 and assembled afterwards using IDBA-Tran v.1.1.1[14] (--mink 19 --maxk 124 --step 5 --min_contig 200 --max_isoforms 1[--max_isoforms 3 in cases of assemblies "*Eu. rowelli* slime gland: endpieces", "*Eu. rowelli* all except gut", and "*P. hitoyensis* all except gut"]). Completeness of tissue-specific transcriptome assemblies were assessed using the metazoan dataset of BUSCO v.5.4.4[15] in transcriptome mode. Obtained sequenced raw reads were submitted to SRA archive and transcriptome assemblies to TSA archive of GenBank, respectively (see **Table S3** for assembly statistics and accession numbers).

Cloning of phosphonate protein genes from cDNA
Transcripts obtained from transcriptomic analyses were used to design gene specific primers for each phosphonate gene of interest (*PEPm, Ppd, AEPt*) of both velvet worm species studied (*Eu. rowelli*: PEPm_forward 5'-atgtgtcaacgtggctca, PEPm_reverse 5'-ttactgtttggcaggcaa, Ppd_forward 5'-atggcctgttgtaaaaaata, Ppd_reverse 5'-tcagtgaatagcaagaaaatg, AEPt_forward 5'-atgaacaacatggaaacatca, AEPt_reverse 5'-ttatttcacaggtaatttaatattc; *P. hitoyensis:* PEPm_forward

5'-atgtggcaacgtggctc, PEPm_reverse 5'-ttaattaggtaaatatttcttctcg, Ppd_forward 5'-atgacgtcttctatcaaacca, Ppd_reverse 5'-ttagttaattgcaagatagtgc, AEPt_forward 5'-atgaaattatctattgtaagaacaa, AEPt_reverse 5'-ttatactattggtaatttaatattcatt). First strand cDNA syntheses were performed from total RNA of both species using SuperScript™ IV First-Strand Synthesis System (Thermo Scientific). After amplification using Phusion™ Plus Green PCR Master Mix (Thermo Scientific) and custom primers (Thermo Scientific), genes were ligated into pJET1.2/blunt vectors using CloneJET PCR Cloning Kit (Thermo Scientific) and transformed into competent *E. coli* TOP10 cells. Resulting recombinant clones were amplified by colony PCR, sequenced (Sanger sequencing, Eurofins Genomics Europe Sequencing GmbH, Konstanz, Germany) and submitted to GenBank (see **Table S6** for accession numbers and deduced protein sequences).

Trypsin digestion of slime samples

Briefly, dry slime was slowly solubilized in a buffer containing 6M guanidine, 500 mM Tris-HCl (pH 8.0) and 3 mM DTT. The mixture was heated at 37 °C for an hour before adding 50 mM ammonium bicarbonate and 1 mM calcium chloride solution to lower guanidine concentration to 1M, finally adjusting pH for best trypsin activity. Protein:protease ratio was set to 20:1 with final protein concentration of 0.3 mg/mL. Digestion was initiated and kept at 37 °C overnight. Digested slime was further separated based on charges using SP (cation exchange) and Q (anion exchange) sepharose peptide ion-exchange columns (Cytiva columns)[16].

Bligh and Dyer lipid extraction

To determine the total lipid composition, we used an adapted protocol of the standard Bligh and Dyer's method as previously reported[17]. Typically, 300 µL of native slime were further diluted with 300 µL of 0.2 M KCl to prevent fiber formation while improving subsequent phase separation. Considering the total amount of water, methanol and chloroform were added to reach a final chloroform:methanol:water molar ratio of 55:35:6. The sample was then sonicated using both bath and probe sonicators (5 cycles of 30 s on ice using 5 inches long probe). The sample was then allowed to phase separate overnight before centrifuging it at 10'000 g for 1 hour to allow best phase separation despite some fiber formation that appeared during sonication.

Additional text highlighting differences between the two species of velvet worms

The slime of *Eu. rowelli* contains a high phosphonate/phosphate [Phn:Pho] ratio (1.00:0.06), while that of *Ep. barbadensis* has only phosphonates (**Figure S3**). This contrasts with the tissue measurements, which exhibit a Phn:Pho ratio of about 1:4 for both species. This finding is consistent with velvet worms having dedicated organs producing concentrated phosphonate-rich molecules. According to ^{31}P spectra peak splitting values in *Eu. rowelli* and *Ep. barbadensis* (140 to 160 Hz), the different resonances detected are not due to one phosphonate atom with surrounding coupling atoms but more likely represent different phosphonate modifications or different phosphonates involved in a phosphonate-polymer in the slime. Therefore, *Eu. rowelli* and *Ep. barbadensis* possibly share similar phosphonates but with different patterns or surrounding molecules. In addition to different phosphonate NMR peaks, the slime of *Ep. barbadensis*, which has no phosphate and overall lower amount of phosphorus despite higher protein concentration, requires lower forces to initiate fiber formation and is harder to resuspend. These mechanical differences combined with the compositional differences may suggest that phosphonates play an important role in determining the mechanical properties of slime; however, their functional role must be further investigated.

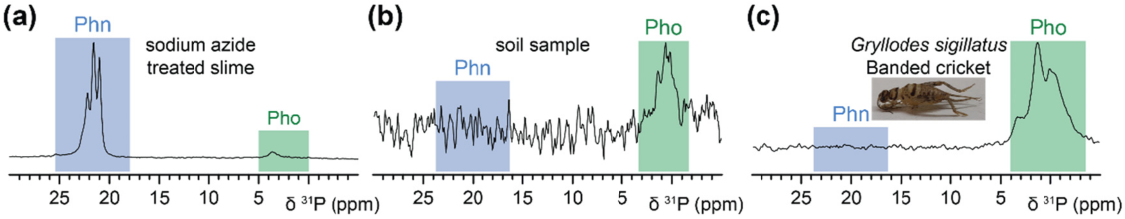

Figure S1. Control for bacteria and environment contamination of the slime samples. (**a**) ^{31}P ssNMR of *Eu. rowelli* slime treated with sodium azide, preventing bacteria growth and contamination during transportation. (**b**) ^{31}P ssNMR of soil used for maintenance of the velvet worms shows usual organic and inorganic phosphorus but no phosphonates. (**c**) ^{31}P ssNMR of ground and hydrated banded cricket (*Gryllodes sigillatus*) – the species used to feed the velvet worms. The spectra show different phosphate molecules but again no phosphonates. ^{31}P ssNMR analyses of control samples confirm that phosphonate moieties are naturally present in velvet worm slime and exclude the possibility of bacterial (**a**), environmental (**b**) or food (**c**) contamination. All spectra were recorded at a field of 9.4 T (400 MHz) with a MAS frequency of 10 kHz using a Bruker 4 mm double resonance probe.

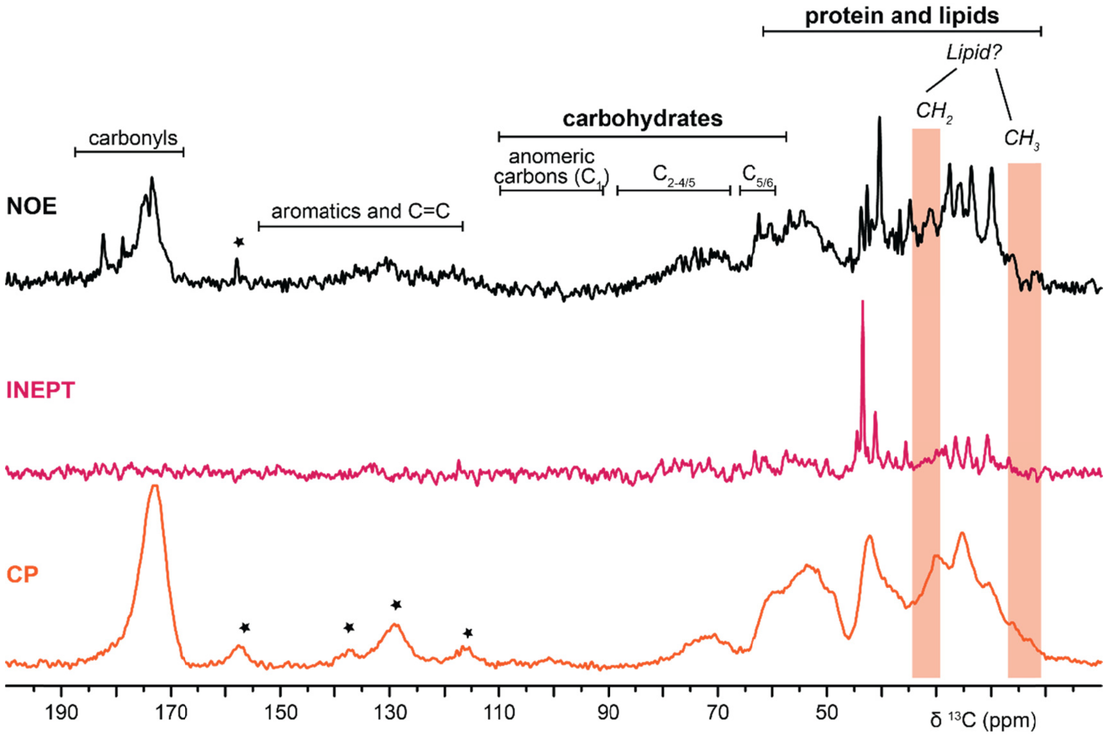

Figure S2. Mobility selective ^{13}C NMR spectra of *Eu. rowelli* native aggregated slime fibers. The complexity of slime can partially be addressed by filtering its components according to their mobility. In biological samples for instance, lipids, peptides and oligosaccharides are expected to be highly mobile, while starch, large/structured/insoluble proteins or mineralized matrices are expected to be more rigid. Using the Nuclear Overhauser Effect (NOE) magnetization transfer, overall slime composition can be semi- quantitatively probed, regardless of the molecular motion. The resulting spectrum (**black**) provides a semi-quantitative description of the slime and confirms the overall slime composition with mostly proteins and very small quantities of glycans. No lipids were detected in the samples even using the Insensitive Nuclei Enhancement by Polarization Transfer (INEPT) pulse sequence (**magenta** spectrum), which is sensitive to mobile molecules and therefore known to be an efficient way to detect lipids[18]. Characteristic lipid resonances at ~15 ppm and ~30 ppm corresponding to CH_3 and CH_2 of lipid acyl chains are highlighted in **orange**. A lipid content below the detection limit of our ^{13}C NMR approach is in line with recent reports of a very low lipid amount (<1%) in slime[19-21]. Using 1.5 ms cross polarization (CP) (**orange** spectrum), which enhances molecules with low mobility, we detected overall broader lines characteristic of solids but no specific molecules that would differ in mobility from the rest of the slime. Stars represent spinning sidebands. All spectra were recorded following the methods of Poulhazan *et al.*[22].

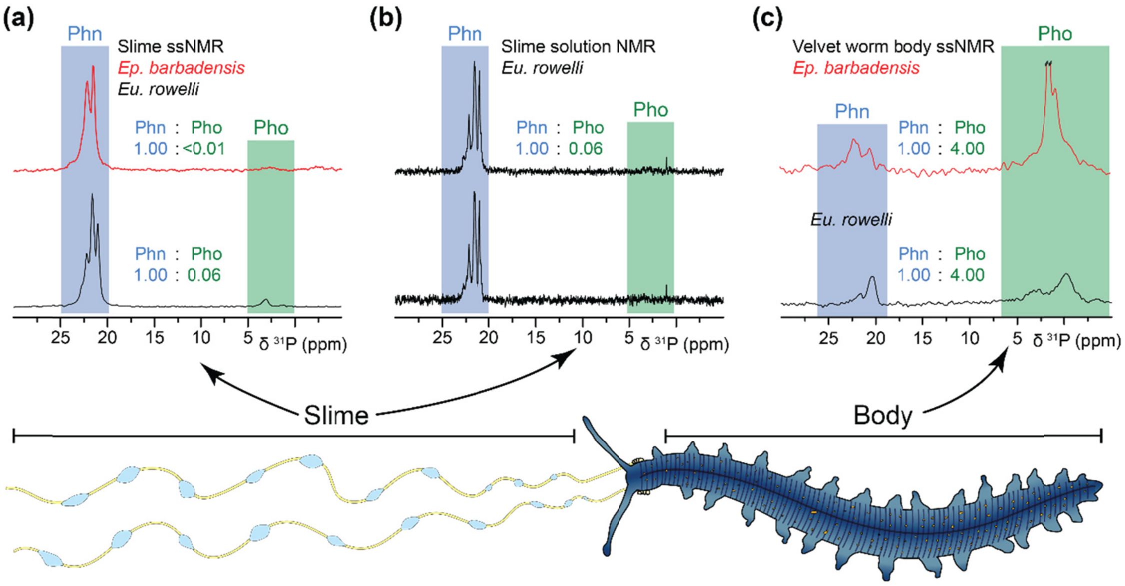

Figure S3. ^{31}P NMR to detect phosphonates in slime and tissues of both velvet worm species. (**a**) ^{31}P ssNMR analysis of different slime fiber aggregates show very similar spectra except for limited amounts of phosphate (Pho) in *Ep. barbadensis* and for minor unexplored differences in the phosphonate (Phn) region. (**b**) Solution NMR spectra of diluted slime in D_2O show very similar ^{31}P resonance patterns to those observed in the fibers, the improved resolution is likely due to molecular tumbling. (**c**) Different velvet worm tissues can be probed by ssNMR, simply filling the ssNMR rotor with the intact tissue. Much lower phosphonate intensity was detected in the bodies of both velvet worm species. Spectra in panels (a) and (c) spectra were recorded at a field of 9.4 T (400 MHz) with a MAS frequency of 10 kHz, while panel (b) spectra were recorded at a field of 14.1 T (600 MHz).

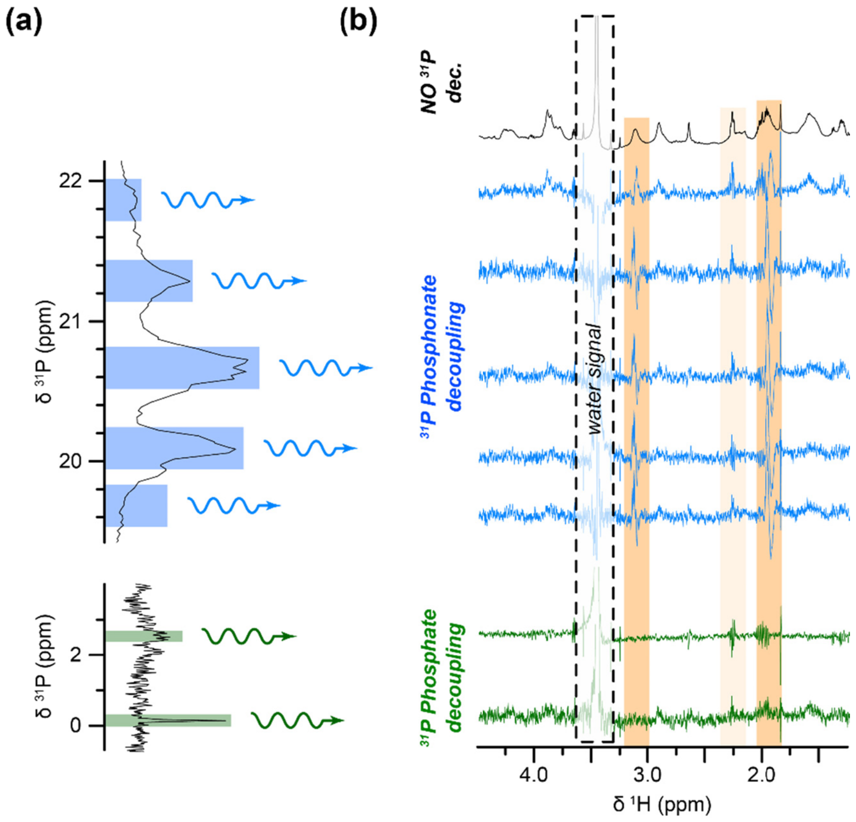

Figure S4. Frequency-specific ^{31}P decoupled ^{1}H solution NMR spectra.
To investigate ^{1}H-^{31}P contacts, we recorded 1D ^{1}H spectra with resonance-specific ^{31}P decoupling corresponding to either the phosphonate or the phosphate groups. (**a**) ^{31}P solution NMR spectra (of native slime diluted in D_2O) enabled us to specify the ^{31}P decoupling frequencies to be selectively decoupled in ^{1}H spectra. Phosphonates appear between 20 ppm and 22 ppm while phosphates are in the 0–2 ppm range. (**b**) The difference between the non-^{31}P-decoupled ^{1}H spectrum (black spectrum, top) and the selectively decoupled spectrum allowed us to identify which protons are coupled to a specific ^{31}P resonance. The decoupling of phosphonate frequencies (**orange** spectra) has a significant effect on ^{1}H spectra compared to phosphate decoupling (**green** spectra). These results suggest that ^{1}H at ~1.97 ppm and ~3.02 ppm chemical shifts are coupled with the phosphonate ^{31}P atoms in *Eu. rowelli* slime (**Table S1**), which is in line with the 2-AEP motif in the slime. Spectra were recorded at room temperature and a field of 14.1 T (600 MHz). Selective ^{31}P decouplings were obtained by continuous wave irradiation of the phosphonate or phosphate resonance at low power (radiofrequency field of 385 Hz) during the acquisition.

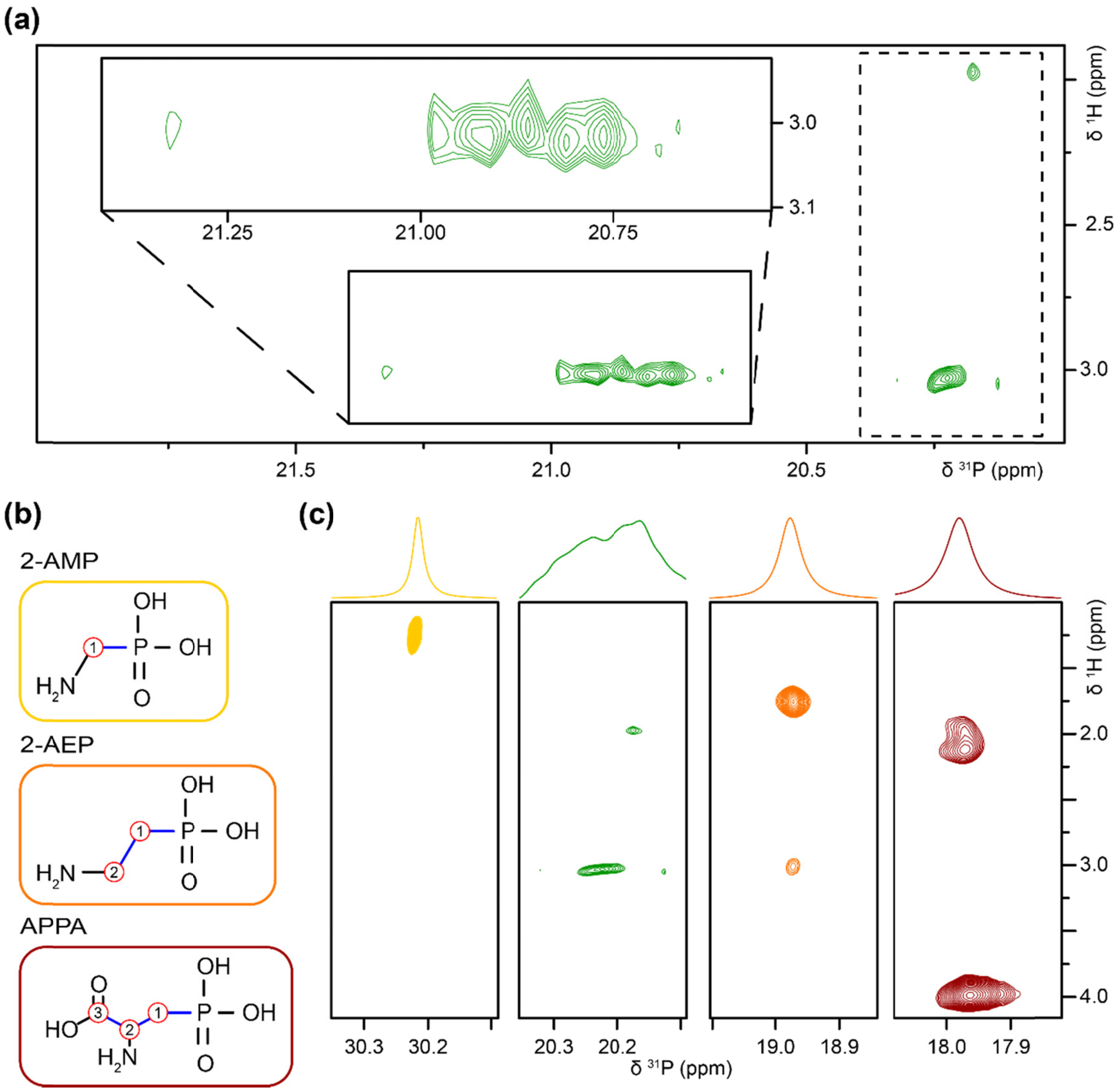

Figure S5. 2D ^{1}H-^{31}P hetero TOCSY for coupling in phosphonate standards and slime. (**a**) ^{1}H-^{31}P 2D TOCSY (Total Correlation Spectroscopy[2]) solution NMR spectra allowed the detection of correlations between resonances at 20.5–21.5 ppm for ^{31}P and 3.0 ppm and 2.0 ppm for ^{1}H in native slimes diluted in D_2O. (**b**) Molecular representation of the phosphonate standards used to identify phosphonates in native slime. The standards are aminomethylphosphonic acid (AMP), 2-aminoethylphosphonic acid (2-AEP) and phosphonoalanine (DL-2-amino-3-phosphonopropionic acid or APPA) all found in natural phosphonates. (**c**) Zoom-in on 2D ^{1}H-^{31}P TOCSY spectra of phosphonate standards and *Eu. rowelli* native slime allowed us to identify the similar proton chemical shift of 2-AEP (at 1.9 ppm and 3.1 ppm) and the slime (at ppm 2.0 and 3.0 ppm) compared to other standards with different ^{1}H, or even ^{31}P (for AMP), chemical shifts. ^{13}C, ^{1}H and ^{31}P chemical shift assignments can be found in **Table S1**. Spectra were recorded at room temperature and a field of 14.1 T (600 MHz) following our previously published procedure[3].

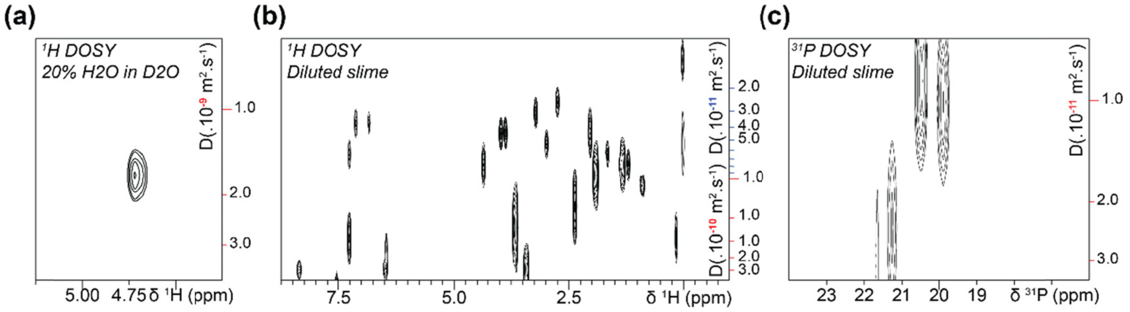

Figure S6. ^{1}H and ^{31}P DOSY of *Eu. rowelli* slime locate phosphonates in large objects. Diffusion Ordered Spectroscopy (DOSY) experiments are used to separate molecules according to their diffusion coefficients which can be read on the vertical axis. (**a**) The diffusion coefficient of water is on the order of 10^{-9} $m^2{\cdot}s^{-1}$. (**b**) The proton signals of most molecules in native slime are indicative of diffusion coefficients between 10^{-10} $m^2{\cdot}s^{-1}$ and 10^{-11} $m^2{\cdot}s^{-1}$, therefore up to two orders of magnitude slower than water. (**c**) Finally, the ^{31}P signals of phosphonate atoms are characterized by the smallest diffusion coefficients which are consistent with their presence in large proteins or even nanoglobules. These results suggest that in the slime, non-phosphonated proteins are in solution within relatively small soluble objects while phosphonated molecules are linked to slowly diffusing large molecules or aggregates. The diffusion dimension was obtained by incrementing the gradient strength linearly between 1.8 $G{\cdot}cm^{-1}$ and 54 $G{\cdot}cm^{-1}$ in 12 increments. The diffusion delays were 100 ms to 200 ms and the gradient duration 4 ms.

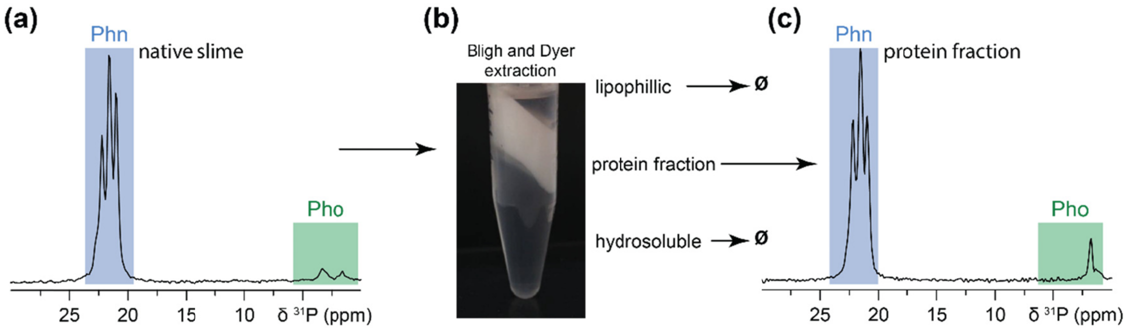

Figure S7. Lipid extraction from slime demonstrates phosphonates in protein fraction. Bligh and Dyer lipid extraction was performed on *Eu. rowelli* slime to separate it into hydrophobic components, water soluble molecules, and a protein interface between the two phases, enabling analysis of the different fractions for phosphate (Pho) and phosphonate (Phn) content. (**a**) NMR spectra of diluted native slime prior to (**b**) Bligh and Dyer lipid extraction. The resulting fractions (from top to bottom: lipophilic, protein interface and hydro soluble) were analyzed by solution NMR and GC-MS, showing a non-detectable by NMR/GC-MS lipid/fatty acid amount in the lipophilic fraction. (**c**) NMR measurements revealed the absence of a Pho signal in the lipophilic and hydrosoluble fractions, whereas analysis of the protein fraction showed a Phn signal very similar to that of the native slime. Measurements of the protein fraction clearly show that phosphate and phosphonates are strongly associated with protein molecules. Results of ^{31}P NMR on these three phases indicate that the phosphonates are located within the protein-rich interface, excluding the possibility that the phosphonate is associated with lipids or small organic or hydro soluble molecules.

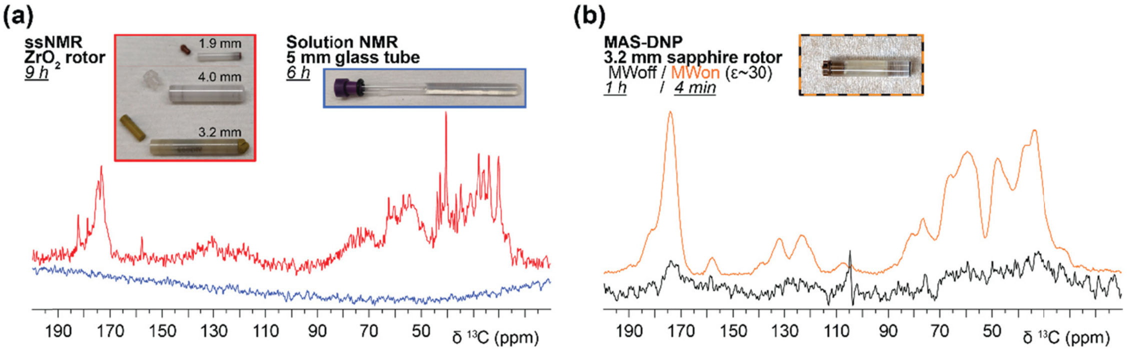

Figure S8. Sensitivity of NMR experiments and MAS-DNP enhancement.
(**a**) The slime of *Eu. rowelli* diluted in D_2O allowed the detection of ^{31}P and ^{1}H in solution but not that of ^{13}C due to limited sensitivity and the low natural abundance (1.1%) of this isotope (**blue** spectrum). However, ^{13}C ssNMR enabled the analysis of different quantities of highly concentrated fiber aggregates samples (~20 mg, ~50 mg and ~80 mg in 1.9 mm, 3.2 mm and 4 mm rotors, respectively). Thus, overnight acquisition provided ^{13}C NOE semi-quantitative spectra with sufficient signal intensity (**red** spectrum, acquired at 14.1 T). (**b**) MAS-DNP increases the intensity of the ^{13}C signal by more than an order of magnitude (~30 times), providing a 1D ^{13}C spectrum of natural abundance samples after only 4 min of acquisition (**orange** spectrum, acquired at 14.1 T), which is not possible without DNP even at low temperature (**black** spectrum). Photographs illustrate typical ssNMR rotors used to study parts of velvet worm body and aggregates of slime fibers, adapted 5 mm solution NMR for viscous and fiber forming slime, and 3.2 mm microwave transparent sapphire rotor for MAS-DNP experiments.

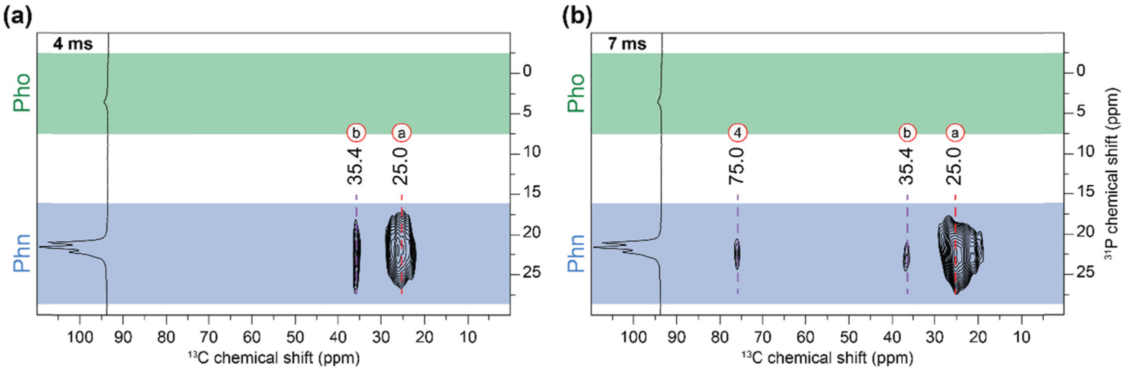

Figure S9. ^{13}C-^{31}P contacts as detected by 2D MAS-DNP on native slime in *Eu. rowelli*. 2D ^{13}C-^{31}P spectra at both 4 ms (**a**) and 7 ms (**b**) CP mixing times confirmed that 1D double ^{1}H-^{31}P-^{13}C CP peaks (**Figure S10a**) arise from contacts with the phosphonate (Phn) in the slime (chemical shift between 16–28 ppm and not from phosphate (Pho). Note that 2D experiments are less sensitive; therefore, lower-intensity peaks were not detected (for instance the 63.3 ppm and 44.4 ppm peaks). All spectra were recorded at 100 K on a 600 MHz MAS-DNP spectrometer using a wide-bore HXY probe and an MAS frequency of 8 kHz.

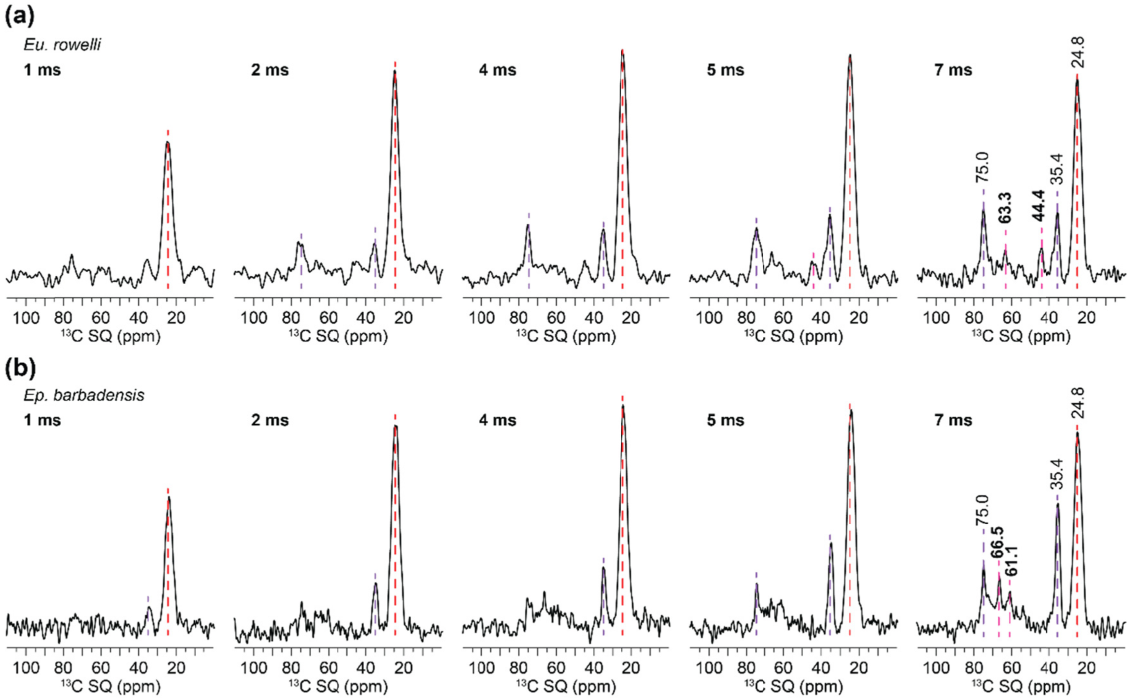

Figure S10. Comparison of MAS-DNP ^{31}P-^{13}C build-up spectra of slimes from both species. Comparison of (**a**) *Eu. rowelli* and (**b**) *Ep. barbadensis* ^{31}P-^{13}C cross-polarization (CP) spectra at different contact time, enabled by a ε~40 times DNP enhancement. Short contact times show only directly linked atoms (at 24.8 ppm). Longer CP contact times progressively allow detection of carbon atoms further away from phosphonates: ^{31}P atoms with resonances at 35.4 ppm followed by those at 75.0 ppm. Finally, resonances at 63.3 ppm could be detected exclusively in *Eu. rowelli*, which corresponds to a ~3.2 Å distance according to DFT calculations. MAS-DNP build-up combined with other methods allowed us to propose a final molecular model (see **Figure 3d** and **Figure S14**). Plot of the *Eu. rowelli* slime build-up can be found in **Figure 3b**. All spectra were recorded at 100 K on a 600 MHz MAS-DNP spectrometer using a custom-made HXY MAS-DNP probe and an MAS frequency of 8 kHz.

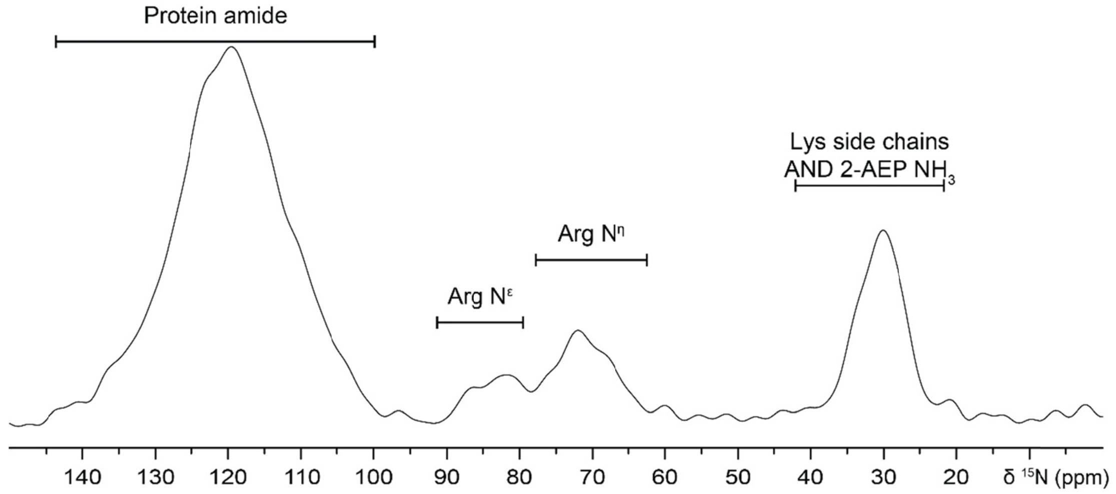

Figure S11. MAS-DNP natural abundance ^{15}N for protein and 2-AEP identification.
1D ^{15}N spectra of *Eu. rowelli* slime allowed detecting the protein backbone as well as amino acid side chains such as the characteristic arginine N^{η}. An intense peak at ~30 ppm is usually assigned to lysine sidechains but could also arise from 2-AEP amine. All spectra were recorded at 100 K on a 600 MHz MAS-DNP spectrometer using a custom-made HXY MAS-DNP probe and an MAS frequency of 8 kHz.

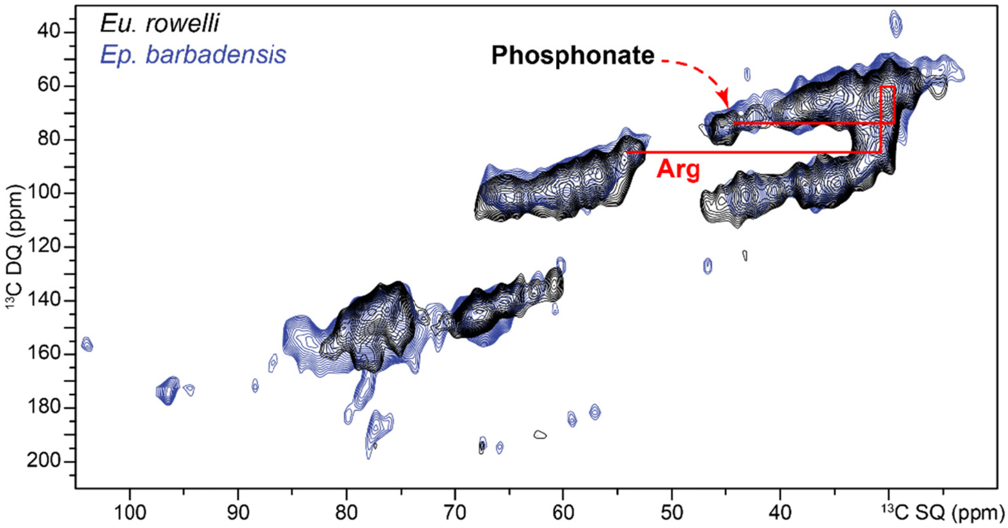

Figure S12. ^{13}C-^{13}C MAS-DNP Double Quantum/Single Quantum correlations in the slime. Comparison of *Eu. rowelli* (**black**) and *Ep. barbadensis* (**blue**) ^{13}C-^{13}C SPC-5[22, 23] correlation spectra revealed only a few differences, providing evidence for similar overall amino acid content. Nevertheless, peaks at ~96 ppm and ~105 ppm are only present in *Ep. barbadensis*, corresponding to glycan anomeric carbons. Note that the signal from *Eu. rowelli* and *Ep. Barbadensis'* slimes were respectively enhanced using AMUPol and AsymPol-POK radicals. Thus, the observed differences can be either due to a higher glycan content in *Ep. barbadensis* slime or, more likely, to a more efficient enhancement from the AsymPolPok radical (~30 *versus* ~50 times enhancement for AMUPol *vs.* AsympPol-POK). Inter-molecular contacts between phosphonates and a carbon resonating at 44.4 ppm carbon were detected in *Eu. rowelli*. This carbon is here shown to be correlated with frequencies at 54.1 ppm, 30.7 ppm and 29.5 ppm, likely corresponding to arginine chemical shifts (see spin system in **red**). All spectra were recorded at 100 K on a 600 MHz MAS-DNP spectrometer using a custom-made HXY MAS-DNP probe and 8 kHz MAS.

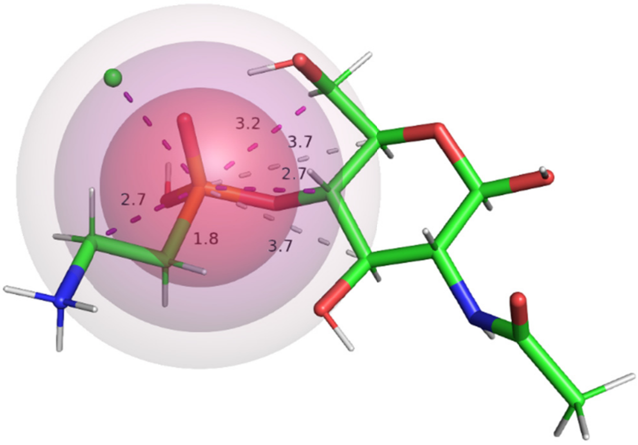

Figure S13. DFT calculated 3D structure representation of the phosphonate in the slime. The structure of the molecule was calculated using DFT approach using r2SCAN-3c. The small green bead represents a hypothesized intermolecular contact between phosphonates and arginine sidechains. Spheres around the phosphonate phosphorous atom correspond to distances of 1.8 Å (red), 2.7 Å (purple) and 3.2 Å (grayish pink).

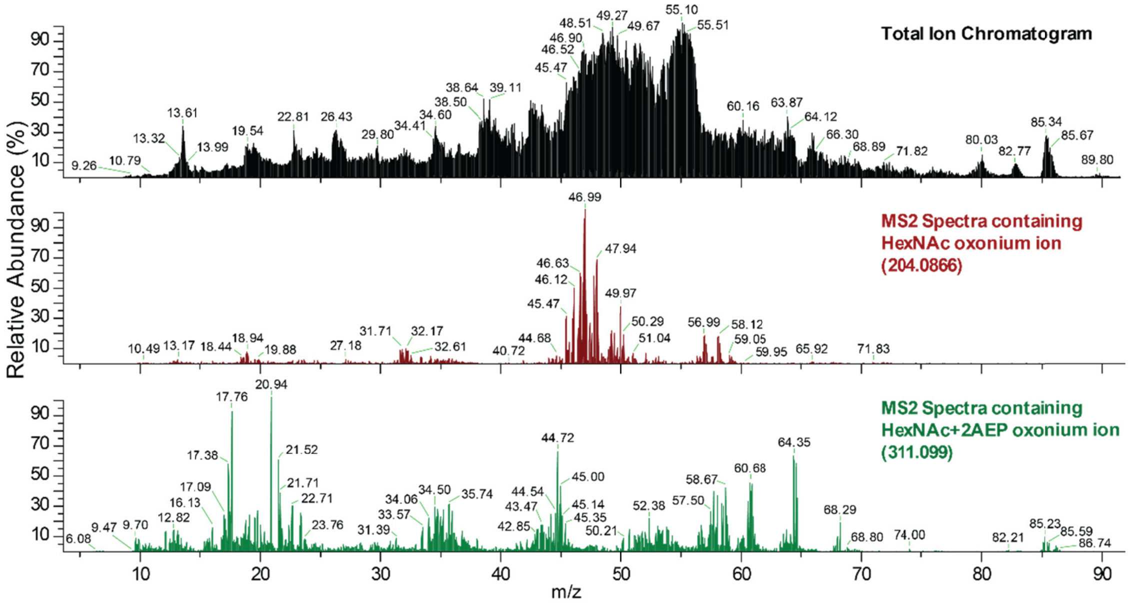

Figure S14. HCD-MS/MS chromatogram of peptide with 2-AEP-glycan modification.
Eu. rowelli slime oxonium ion *m/z* traces used to identify 2-AEP modified sugars. Traces include total ion chromatogram (**black**), HexNAc oxonium ion (*m/z* 204.0866, **red**) and HexNAc+ AEP oxonium ion (*m/z* 311.099, **green**).

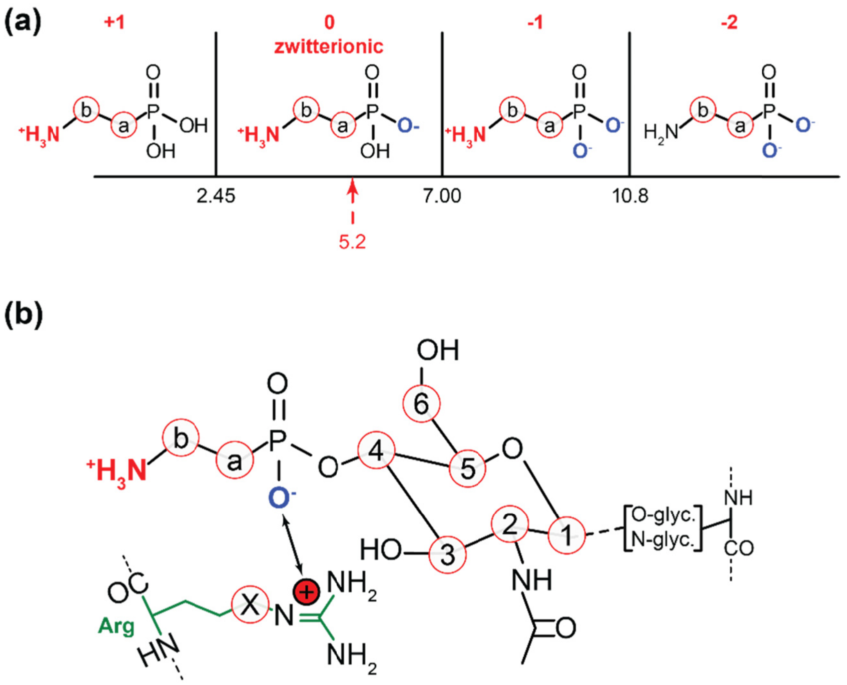

Figure S15. 2-AEP charge and corresponding phosphonates in velvet worm slime.
(**a**) 2-AEP representation as a function of pH (given the pKas of the molecule) suggests that 2-AEP will likely be zwitterionic at pH 5.2, the native pH of slime in *Eu. rowelli*. (**b**) Extrapolating this behavior to the proposed phosphonate pattern decorating slime proteins in *Eu. rowelli*, interaction between HMW phosphonates and the arginine side chain might be possible through electrostatic interactions that might be important for the formation of slime fiber.

Table S1. ^{31}P, ^{13}C and ^{1}H chemical shift of phosphonate standards and slime.

The assignment of standards and native slime of both studied onychophoran species were detected by solution/solid-state NMR and MAS-DNP.

Full name	^{31}P	$^{13}C_1$	$^{13}C_2$	$^{13}C_3$ (ppm)	$^{1}H_1$	$^{1}H_2$	$^{1}H_N$	^{15}N
aminomethylphosphonic acid (AMP)	33.4	12.0	-	-	1.3	-	7.2	-
2-aminoethylphosphonic acid (2-AEP)	18.9	26.1	35.4	-	1.9	3.1	7.5	33.3
2-aminoethylphosphonic acid + HCl	23.7	24.8	34.4	-	1.9	3.0	-	-
2-amino-3-phosphonopropionic acid (APPA)	17.9	28.0	49.6	171.9	2.2	4.1	7.3	-
Eu. rowelli slime – solution NMR	22.2/21.6/21.0	-	-	-	2.0	3.0	-	-
Eu. rowelli slime – ssNMR/MAS-DNP	22.2/21.6/21.0	24.8	35.4	-	2.0	3.0	-	31.8
Ep. barbadensis slime – ssNMR	22.2/21.5	-	-	-	-	-	-	-

Table S2. Known natural phosphonate moieties. Terrestrial invertebrates with reported phosphonate moieties are highlighted in **red**.

Taxonomic Classification	Species	Molecule	Reference
**Glycolipids and glycoceramides (CAEPn and CMAEPn)**			
Bacteria	gram-negative, obligate aerobic bacterium *Bdellovibrio bacteriovorus*	phosphonolipids	Steiner *et al.* (1973)[26]
Bacteria	marine bacterioplankton species *Prochlorococcus* sp. and SAR11	phosphonoglycoproteins polysaccharide caspule lipids	Acker *et al.* (2020)[27]
Bacteria	predatory bacterium *Bacteriovorax stolpii* (mostly feeding on larger gram-negative, enteric bacteria)	chydroxy-MAEP	Jayasimhulu *et al.* (2007)[28] Watanabe *et al.* (2001)[29]
Bacteria	representatives of Proteobacteria, Bacteroidetes, Firmicutes, Actinobacteria, and Spirochaetes	phosphonolipids/glycans	Yu *et al.* (2013)[30]
Eukaryota: Excavata	parasitic euglenoid *Trypanosoma cruzi*	phosphono glycolipid	Serrano *et al.* (1995)[31]
Eukaryota: Excavata	parasitic euglenoid *Trypanosoma cruzi*	phosphonolipid	Ferguson *et al.* (1982)[32]
Eukaryota: Excavata	parasitic euglenoid *Trypanosoma cruzi*	phosphonolipids/glycans	Yu *et al.* (2013)[33]
Eukaryota: Amoebozoa	several amoeba species	(hydroxy) 2-AEP glycolipid	Korn *et al.* (1973)[34]
Eukaryota: Ciliophora	freshwater ciliate *Tetrahymena pyriformis*	2-AEP-phosphonolipid	Kennedy *et al.* (1970)[35]
Eukaryota: Ciliophora	freshwater ciliates *Paramecium tetraurelia* and *Tetrahymena thermophila*	phosphonolipids/glycans	Yu *et al.* (2013)[30]
Eukaryota: Alveolata	oyster parasite *Perkinsus marinus*	phosphonolipids/glycans	Yu *et al.* (2013)[30]
Eukaryota: Fungi	plant pathogen fungus *Pythium prolatum*	CAEPn	Wassef *et al.* (1977)[36]
Metazoa: Porifera	sea sponge *Placospongia* sp.	phosphoiodyn	Kim *et al.* (2013)[37]
Metazoa: Cnidaria	sea anemone *Anthopleura elgantissima*	CAEPn	Hori *et al.* (1993)[38]
Metazoa: Cnidaria	sea anemone *Nematostella vectensis*	phosphonolipids/glycans	Yu *et al.* (2013)[33]
Metazoa: Cnidaria	night-light jellyfish *Pelagia noctiluca* (Scyphozoa)	sphingophosphonolipid	Kariotoglou & Mastronicolis (2003)[39]
Bilateria: Echinodermata	sea urchin *Anthocidaris crassispina*	CAEPn	Hori *et al.* (1993)[38]
Bilateria: Annelida	**megascolecid earthworm *Metaphire hilgendorfi***	CAEPn	Mutsumi *et al.* (1995)[40]
Bilateria: Annelida	**megascolecid earthworm *Metaphire hilgendorfi***	phosphono glycosphingolipid	Sugita *et al.* (1995)[41]
Bilateria: Mollusca	sea hare *Aplysia kurodai*	CAEPn	Araki *et al.* (1986)[42]; (1989)[43]
Bilateria: Mollusca	sea hare *Aplysia kurodai*	sphingophosphonolipid	Yamada *et al.* (1995)[44] Akari *et al.* (1991)[45]
Bilateria: Mollusca	sea hare *Aplysia kurodai*	CAEPn	Satake *et al.* (2012)[46]
Bilateria: Mollusca	freshwater pulmonate snail *Helisoma* sp.	glyco lipids or phosphonoglycoprotein	Miceli *et al.* (1987)[47]
Bilateria: Mollusca	marine vetigastropod snail *Chlorostoma argyrostoma*	polar phosphonolipid	Zhukova *et al.* (2014)[48]
Bilateria: Mollusca	marine vetigastropod snail *Chlorostoma argyrostoma*	glycosphingolipid	Matsubara *et al.* (1986)[49]
Bilateria: Mollusca	marine vetigastropod snail *Turbo cornutus*	CMAEPn	Hayashi *et al.* (1989)[50]
Bilateria: Mollusca	marine patellogastropod snail *Lottia gigantea*	phosphonolipids/glycans	Yu *et al.* (2013)[33]

Bilateria: Mollusca	several marine snails<br>sea snail *Turbo cornutus*	CMAEPn	Matsubara *et al.* (1990)[51]<br>Matsubara *et al.* (1981)[52]
Bilateria: Mollusca	scallop *Hinnites giganteum*	CAEPn	Hori *et al.* (1993)[38]
Bilateria: Mollusca	squid *Ommastrephes bartramii*	CAEPn	Saito *et al.* (2012)[53]
Bilateria: Mollusca	octopus *Ledone moschata*, cuttlefish *Sepia officinalis*, and squid *Todarodes sagittatus*	CAEPn	Sinaglou *et al.* (2004)[54]
Bilateria: Mollusca	chocolate-band snail *Eobania vermiculata*, and Mediterranean mussel *Mytilus galloprovincialis*	sphingophosphonolipid	Kariotoglou & Mastronicolis (2003)[39]
Bilateria: Arthropoda	antarctic krill *Euphausia superba*	CMAEPn	Itonori *et al.* (1991)[55]
Bilateria: Arthropoda	shore shrimp *Farfanteenaeus aztecus*	CAEPn or CMAEPn	Shimomura *et al.* (1983)[56]
**Glycans**			
Bacteria	obligate anaerobic gram-negative bacillus *Bacteroides fragilis*	2-AEP-glycan antigen<br>phosphonated capsular polysaccharide<br>GlcNAc/Gal/Qui	Baumann *et al.* (1992)[57]
Bacteria	obligate anaerobic gram-negative bacillus *Bacteroides fragilis*	phosphono polysaccharide B<br>capsular polysachharide	Coyne *et al.* (2000)[58]<br>Tzianabos *et al.* (1992)[59]<br>Baumann *et al.* (1992)[57]
Bacteria	several representatives	2-AEP-glycan on cell surface	Rice *et al.* (2019)[60]
Bacteria	gram-positive soil bacteria *Glycomyces* sp. and *Stackebrandtia nassauensis*	phosphono glycan<br>phosphono pullulan like	Yu *et al.* (2014)[61]
Bilateria: Nematoda	**free-living nematode *Caenorhabditis elegans* (abundant in microbe-rich environments, especially decomposing plant matter)**	phosphono N-glycan	Paschinger *et al.* (2008)[62]
Bilateria: Platyhelminthes	**dog tapeworm *Echinococcus granulosus* (parasitic flatworm, which causes hydatid disease in humans)**	phosphono glycan epitope	Paschinger *et al.* (2012)[63]
Bilateria: Mollusca	marine neogastropod snail *Volvarina rubella*	(methyl) 2-AEP-Man/Gal	Eckmair *et al.* (2016)[64]
Invertebrates and protozoans		phosphono N-glycans	Paschinger *et al.* (2016)[65]
**Glycoproteins**			
Bacteria	marine bacterioplankton species *Prochlorococcus* sp. and SAR11	phosphono glycoproteins<br>polysaccharide caspules<br>Phosphonolipids	Acker *et al.* (2020)[27]
Eukaryota: Excavata	parasitic euglenoid *Trypanosoma cruzi*	AEP-lipopeptidophosphoglycan<br>LPPG (ceramide tail)<br>cell-surface glycol conjugate<br>2-AEP glycan	Previato *et al.* (1990)[66]
Eukaryota: Excavata	parasitic euglenoid *Trypanosoma cruzi*	glycosylphosphatidylinositol membrane-anchored phosphono glycoprotein	Previato *et al.* (1995)[67]
Eukaryota: Excavata	parasitic euglenoid *Trypanosoma cruzi*	AEP-lipopeptido phosphoglycan<br>cell-surface glycoconjugate	De Lederkremer *et al.* (1991)[68]
Eukaryota: Ciliophora	freshwater ciliate *Tetrahymena pyriformis*	AEP-macromolecules	Hilderbrand *et al.* (1983)[69]
Metazoa: Cnidaria	sea anemone *Metridium dianthus*	phosphonated protein	Quin *et al.* (1965)[70]

Metazoa: Cnidaria	marine scyphozoan jellyfish *Aurelia aurita*	mucin decorated with O-glycan	Urai *et al.* (2009)[71]
Metazoa: Cnidaria	sea anemones *Anthopleura xanthogrammica* and *Metridium senile*	AEP-macromolecules	Hilderbrand *et al.* (1983)[69]
Eukaryota: Ciliophora<br>Metazoa: Cnidaria<br>Bilateria: Mollusca	freshwater ciliate *Tetrahymena pyriformis*<br>sea anemones; mussels	phosphonoalanine bound to glycoprotein?	Horsman *et al.* (2017)[72]<br>Quin *et al.* (2001)[73]
Bilateria: Arthropoda	**migratory locust *Locusta migratoria* (terrestrial orthopteran insect)**	AEP-glycoprotein apolipoprotein III	Hard *et al.* (1993)[74]
	**Small organic molecules**		
Achaea	marine, aerobe, chemoautolithotroph, rod-shaped archaeon *Nitrosopumilus maritimus*	methyl 2-AEP	Metcalf *et al.* (2012)[75]
Bacteria	antibiotic-producing species *Streptomyces lavendulae, S. fradiae*, and *S. wedmorensis*	fosmidomycin<br>fosfomycin	Shiraishi *et al.* (2021)[76]
Bacteria	antibiotic-producing species *Streptomyces viridochromogenes*, and gram-negative species *Pseudomonas gladioli*	fosfomycin	Nakashita *et al.* (1992)[77]<br>Hidaka *et al.* (1992)[78]
Bacteria	antibiotic-producing species *Streptomyces hygroscopicus* and *S. viridochromogenes*	bialaphos	Hara *et al.* (1991)[79]
Bacteria	antibiotic-producing species *Streptomyces regensis*	cyanohydrin small organic molecule	Cioni *et al.* (2014)[80]
Bacteria	*Actinomyces* sp.	tripeptide K-26	Ntai *et al.* (2005)[81]
Bacteria	several species	diversity of natural phosphonates<br>mostly short peptides as herbicides	Zhou *et al.* (2020)[82]
Eukaryota: Ciliophora	sheep rumen ciliates (first natural phosphonate)	2-aminoethane phosphonic acid	Horiguchi *et al.* (1959)[83]
Bilateria: Arthropoda	marine crab *Allinectes sapidus*	2-AEP	Kleps *et al.* (2007)[84]
Bilateria: Vertebrata	human brain and other tissues	2-AEP	Alhadeff *et al.* (1970)[85]<br>Castronovo *et al.* (1996)[86]
	**Unidentified phosphonate moiety**		
Bacteria	gram-negative phytopathogen *Xanthomonas axonopodis*	unknown	Harrison *et al.* (2014)[87]
Bacteria	plant-associated bacterium *Cupriavidus plantarum*	unknown	Arroyo-Herrera *et al.* (2020)[88]
Eukaryota: Amoebozoa	human parasite *Acanthamoeba castellanii*	unknown 2-AEP	Deslauriers *et al.* (1980)[89]
Eukaryota: Fungi	basidiomycete mushrooms	unknown	Maciejczyk *et al.* (2014)[90] Koukol *et al.* (2008)[91]
Metazoa: Cnidaria	soft coral *Leptogorgia virgulate*	unknown 2-AEP	Shelburne *et al.* (1967)[92]
Bilateria: Mollusca	nudibranch sea snail *Archiodoris* sp.	unknown 2-AEP	Shelburne *et al.* (1967)[92]
Bilateria: Mollusca	**terrestrial pulmonate slug *Meghimatium bilineata***	unknown	Chichibu *et al.* (1993)[93]
Bilateria: Vertebrata	human erythrocytes	hydroxy 2-AEP	Robitaille *et al.* (1988)[94]

Table S3. Sequencing and assembly statistics of tissue-specific transcriptomes from *Eu. rowelli* and *P. hitoyensis*.

Species	Tissue	# of raw read pairs	SRA accession numbers	# of contigs	Contig N50	BUSCO completeness	Assembly accession
Eu. rowelli	Slime gland: endpieces	213,022,328	SRR25779281<br>SRR25779287<br>SRR25779288	101,343	1,347	C:95.0%[S:71.3%, D:23.7%],F:2.8%, M:2.2%,n:954	GKOF00000000
	Slime gland: reservoir	42,979,592	SRR25779282	306,617	475	C:94.2%[S:92.3%, D:1.9%],F:3.7%, M:2.1%,n:954	GKOG00000000
	Slime gland: duct	53,199,986	SRR25779280	50,596	758	C:77.4%[S:76.5%, D:0.9%],F:10.2%, M:12.4%,n:954	GKOH00000000
	Gut	39,669,658	SRR25779279	87,178	1,226	C:96.6%[S:91.4%, D:5.2%],F:2.2%, M:1.2%,n:954	GKOI00000000
	All above, except gut	309,201,906	SRR25779280<br>SRR25779281<br>SRR25779282<br>SRR25779287<br>SRR25779288	362,127	676	C:97.6%[S:63.6%, D:34.0%],F:2.3%, M:0.1%,n:954	GKOQ00000000
P. hitoyensis	Slime gland: endpieces	69,989,779	SRR25779289	48,025	991	C:87.1%[S:84.4%, D:2.7%],F:5.7%, M:7.2%,n:954	GKOJ00000000
	Slime gland: reservoir	74,594,009	SRR25779290	199,147	896	C:97.4%[S:89.4%, D:8.0%],F:1.6%, M:1.0%,n:954	GKOK00000000
	Slime gland: duct	65,993,848	SRR25779285	51,066	1,011	C:87.9%[S:85.6%, D:2.3%],F:6.5%, M:5.6%,n:954	GKOL00000000
	Head	63,081,578	SRR25779284	243,968	780	C:98.2%[S:87.7%, D:10.5%],F:1.2%, M:0.6%,n:954	GKOM00000000
	Salivary gland	56,690,494	SRR25779283	85,893	899	C:94.6%[S:92.7%, D:1.9%],F:2.9%, M:2.5%,n:954	GKON00000000
	Gut	43,799,398	SRR25779286	110,009	1,041	C:96.7%[S:91.7%, D:5.0%],F:1.9%, M:1.4%,n:954	GKOO00000000
	All above, except gut	330,349,708	SRR25779283<br>SRR25779284<br>SRR25779285<br>SRR25779289<br>SRR25779290	394,541	1,379	C:99.1%[S:36.7%, D:62.4%],F:0.7%, M:0.2%,n:954	GKOP00000000

(C) Complete BUSCOs
(S) Complete and single-copy BUSCOs
(D) Complete and duplicated BUSCOs
(F) Fragmented BUSCOs
(M) Missing BUSCOs
(n) Total BUSCO groups searched.

Table S4. Local BLAST of phosphonate biocatalytic enzymes in the spirochete bacterium *Treponema denticola*.

These biocatalytic enzymes were proposed by Bartlett *et al.*[95] and compared against tissue-specific transcriptomes of *Eu. rowelli* (Peripatopsidae) and *P. hitoyensis* (Peripatidae).

Species	Tissue	PEPm Tde1413 (E-value)	Ppd Tde1414 (E-value)	AEPt Tde1415 (E-value)
Eu. rowelli	Slime gland: endpieces	1e-036	2e-098	3e-043
Eu. rowelli	Slime gland: reservoir	2e-036	2e-098	3e-043
Eu. rowelli	Slime gland: duct	5e-037	7e-099	1e-043
Eu. rowelli	Gut	3e-036	2e-098	3e-043
P. hitoyensis	Slime gland: endpieces	2e-040	6e-096	1e-046
P. hitoyensis	Slime gland: duct	2e-040	7e-096	7e-047
P. hitoyensis	Slime gland: reservoir	1e-039	2e-095	8e-047
P. hitoyensis	Head	2e-039	7e-044	3e-046
P. hitoyensis	Salivary gland	3e-040	1e-095	1e-046
P. hitoyensis	Gut	2e-036	1e-095	7e-047

PEPm = Phosphoenolpyruvate mutase
Ppd = Phosphoenolpyruvate decarboxylase
AEPt = 2-Aminoethyl phosphonate transaminase.

Table S5. Best matches of protein BLAST of phosphonate enzymes in velvet worms. The BLAST was performed against NCBI data base, (E-value).

Eu. rowelli PEPm	PEPm *Crassostrea virginica,* XP_022286300.1, (2e-174)
Eu. rowelli Ppd	Ppd *Pecten maximus,* XP_033752776.1, (0.0)
Eu. rowelli AEPt	AEPt *Nymphon striatum,* KAG1666199.1, (0.0)
P. hitoyensis PEPm	PEPm *Crassostrea gigas,* XP_011425742.1, (4e-173)
P. hitoyensis Ppd	Ppd *Pecten maximus,* XP_033752776.1, (0.0)
P. hitoyensis AEPt	AEPt *Nymphon striatum,* KAG1666199.1, (1e-181)

PEPm = Phosphoenolpyruvate mutase
Ppd = Phosphoenolpyruvate decarboxylase
AEPt = 2-Aminoethyl phosphonate transaminase.

Table S6. Deduced protein sequences of cloned phosphonate enzyme genes from *Eu. rowelli* (Peripatopsidae) and *P. hitoyensis* (Peripatidae). GenBank accession numbers are given in the sequence identifiers.

>PEPm_Euperipatoides_rowelli_OR495729
MCQRGSKLIFSSWYFKGLPKCQQWYSSSSTTIKKTTQLKKMLTSSNLEFLMEAHNGISAKIVQEAG
FKGVWASGLSISAQLGVRDSNEASWTQVLEVLEFMSDATDIPILLDADTGYGNFNNARRLIAKLE
DRGIAGACLEDKLFPKTNSLLDGREQPLADIEEFALKIKACKEHQKDADFCVVARVEAFIAGWGLE
EALKRAEAYKNAGADAILMHSKKSDPSDIDAFMKAWNNQAPVVIVPTKYYKTPLQHFKDLNISTII
WANHNLRASVAAMKDITGTIFKEQSLTGVENKIATVKEIFRLQKDEELVEAEKRYLPAKQ*

>PEPm_Principapillatus_hitoyensis_OR495730
MWQRGSKFLFSVYFNKGHVMAARNYTTTPKKTTKLKQMLTSSNLEFLMEAHSGISAKIVEETGFS
GIWASGLSISAQLGVRDSNEASWTQVLEVIEFMSDATSIPILLDADTGYGNFNNARHLIRKLEDRGI
AGACLEDKLFPKTNSLLDDRKQPLASIEEFALKIKACKDYQRDPDFCIVARVEAFIAGWGLEEALK
RAEAYRKAGADAILMHSKKSDPSDIEAFMKAWNNQGPVVIVPTKYYKTPVQNFKELNISTVIWAN
HNLRASVTAMQDVSQTIFKEQSLVNVENKIAPVKEIFRLQNDNELIEAEKKYLPN*

>Ppd_Euperipatoides_rowelli_OR495731
MACCKKYWAALPLARHLIKSRNKNVSRMFSTSSWLCESKLKLTEPEKQVKQQQERNKEAGMMT
ELVRDFLDPAEFFASIQNIGIDFYCGVPDSLLSDFCAYISDRVPKENHIITSNEGSAVALAAGYHLST
GKTSLVYLQNSGLGNAVNPIMSLAVPNVYSIPLLLLIGWRGEPGKRDEPQHRVQGQATPGLLAAL
GIPFQPLPDYQEGAEQALQTAKQHMDSCKGPYALLVKRQTFLPYKLPKPLSEFPLNRESALKVIVD
CLGDRDVIVSTTGMLSRELFEYRVVKDQGHERDFLTVGSMGHASTIAMGIALNKSRRHVICLDGD
GAVIMHMGGLATIGQNAPTNFKHIIINNGVHDSVGGQPTDAGNHETFSFPIIARGCGYKDAWCAIT
EDEIRDGIVRMKKQDGPILMEIKVDKGGRKDLGRPTRTPQENKKDFMHFLAIH*

>Ppd_Principapillatus_hitoyensis_OR495732
MTSSIKPVIKYLIKSTCSKTIGRTLQTSSCLYDSRAKLEHGHSLPEPEQQVQQQQERNKEAGMMTE
LVRDFLDPAEFFSAVQNIGINFYCGVPDSLLSDFCAYVSIHVTKENHIITANEGTAVAMAAGYHLA
TGKSAMVYLQNSGLGNAVNPLMSLAVPNVYSIPLLLLIGWRGEPGKRDEPQHRVQGQATPGLLA
ALGIPFQPLPDYQEGAEQALQTAKQHMDSCKGPYAFLVKRQTFLPYKLPKSLSQFPLNREGALKVI
ADCLGDRDVVVSTTGMLSRELFEHRAIKNQGHERDFLTVGSMGHASTIAMGISLYKPRRQVFCLD
GDGSVIMHMGSLATIGQNGPSNFKHIIINNGVHDSVGGQPTDAANHENFLFPVIARGCGYKDAWV
AVTEEEIKEGIIKMKKQEGPVMMEIKVDKGGRKDLGRPTRTPQENKADFMHYLAIN*

>AEPt_Euperipatoides_rowelli_OR495733
MNNMETSIFRSFLRPTILFKPHIFSSRIPVKFLTKSMKQLRKITGTGNLFQGEKKLFTPGPLGVSATT
KEAMLRDVGSRDIEFIQLIKYIRRQLLIIAGVSTNSYTAIPLQGSGTYAVEAVLLTSTPLANGRVLILE
NGAYGKRMKKICELANLKHEILSFNENEQVDVGKVETILKHDANFTTVSIVHCETSSGVINPIEKIG
QLVKRYTTDTAYFVDAMSSFGAIPIDMETSHIDYLVSSANKCIQGVPGFSYAIARLDHLLNCKGNC
HSLSLDLVDQHLALEANGQFRFTPPTHAMLAFNQALIEFNHEGGVKGRAIRYQENRLVIRDGMRK
LGFKELLDDSHKGYIITSFHFPKDNNFKFEEFYNRLNEMGQVIYPGKVTNADCFRIGNIGHLFPEDM
KHLLSCIEIVCQQMNIKLPVK*

>AEPt_Principapillatus_hitoyensis_OR495734
MKLSIVRTITRSFISYKLNSYLLPTRFSQMFPLVRTLTSSGPSADIPEGKKLFTPGPLCVSSTVKEAML
RDLGSRDVEYIEVIKFIRQELLNIAGVSSNNFTAIPLQGSGTYAVEAVLITATSKQNGRVLILENGAY
GKRMKKICELAKLRHECISFNEDEVVNINKVEAILKHDSNFTVVGIIHCETSSGIINPIEKVGQLIKK
YTQAVYFVDAMSSFGAVPINMEDCNIDYLVSSANKCIQGVPGFSYAIARLDHLINCKGNCHSLSLD
LVDQHESLEKNGQFRFTPPTHSMLAFKQALTEFYEEGGVHGRAERYEENRGILREGMKKMGFKEF
LDESHQGYIITSYNFPKDSNFKFEQFYNLLNEMDQVIYPGKVLQADCFRIGNIGNIFPEDMKNLLLC
IEKVCQKMNIKLPIV*

Table S7. Comparison of slime and reported glycophosphonate chemical shifts.
Assignment using solution NMR, ssNMR and MAS-DNP of the slime of *Eu. rowelli* are compared to glycophosphonate reported by Baumann *et al.*[58] in bacteria capsule.

		$^{13}C/^{1}H$ chemical shifts	
		Slime (ppm)	*lit.* [58] (ppm)
2-AEP	a	24.8/2.0	27.0/2.1
	b	35.4/3.0	37.2/3.4
Intermolecular (Arginine?)	X	44.4	
N-acetyl glycan	1		104.7
	2		57.2
	3		76.4
	4	75.0	73.3/4.2
	5		77.1/3.6
	6	63.3	62.2/3.9

Movie S1. Slow motion video of slime ejection by a living specimen of *Ep. barbadensis*.
See separate file.

References

1. Arnold, A. A.; Genard, B.; Zito, F.; Tremblay, R.; Warschawski, D. E.; Marcotte, I., Identification of lipid and saccharide constituents of whole microalgal cells by ^{13}C solid-state NMR. *Biochim. Biophys. Acta - Biomembr.* **2015**, *1848* (1, Part B), 369–377.
2. Balsgart, N. M.; Mulbjerg, M.; Guo, Z.; Bertelsen, K.; Vosegaard, T., High throughput identification and quantification of phospholipids in complex mixtures. *Anal. Chem.* **2016,** *88* (4), 2170–2176.
3. Kumar, K.; Sebastiao, M.; Arnold, A. A.; Bourgault, S.; Warschawski, D. E.; Marcotte, I., *In situ* solid-state NMR study of antimicrobial peptide interactions with erythrocyte membranes. *Biophys. J.* **2022,** *121* (8), 1512–1524.
4. Morcombe, C. R.; Zilm, K. W., Chemical shift referencing in MAS solid state NMR. *J. Magn. Reson.* **2003,** *162* (2), 479–486.
5. Cade-Menun, B. J., Improved peak identification in ^{31}P-NMR spectra of environmental samples with a standardized method and peak library. *Geoderma* **2015,** *257–258*, 102–114.
6. Wishart, D. S.; Bigam, C. G.; Yao, J.; Abildgaard, F.; Dyson, H. J.; Oldfield, E.; Markley, J. L.; Sykes, B. D., ^{1}H, ^{13}C and ^{15}N chemical shift referencing in biomolecular NMR. *J. Biomol. NMR* **1995,** *6* (2), 135–140.
7. Lilly Thankamony, A. S.; Wittmann, J. J.; Kaushik, M.; Corzilius, B., Dynamic nuclear polarization for sensitivity enhancement in modern solid-state NMR. *Prog. Nucl. Magn. Reson. Spectrosc.* **2017,** *102–103*, 120–195.
8. Sauvée, C.; Rosay, M.; Casano, G.; Aussenac, F.; Weber, R. T.; Ouari, O.; Tordo, P., Highly efficient, water-soluble polarizing agents for dynamic nuclear polarization at high frequency. *Angew. Chem. Int. Ed. Engl.* **2013,** *52* (41), 10858–10861.
9. Mentink-Vigier, F.; Marin-Montesinos, I.; Jagtap, A. P.; Halbritter, T.; van Tol, J.; Hediger, S.; Lee, D.; Sigurdsson, S. T.; De Paëpe, G., Computationally assisted design of polarizing agents for dynamic nuclear polarization enhanced NMR: the AsymPol family. *J. Am. Chem. Soc.* **2018,** *140* (35), 11013–11019.
10. Harrabi, R.; Halbritter, T.; Aussenac, F.; Dakhlaoui, O.; van Tol, J.; Damodaran, K. K.; Lee, D.; Paul, S.; Hediger, S.; Mentink-Vigier, F.; Sigurdsson, S. T.; De Paëpe, G., Highly efficient polarizing agents for MAS-DNP of proton-dense molecular solids. *Angew. Chem. Int. Ed.* **2022,** *61* (12), e202114103.
11. Lilly Thankamony, A. S.; Wittmann, J. J.; Kaushik, M.; Corzilius, B., Dynamic nuclear polarization for sensitivity enhancement in modern solid-state NMR. *Prog. Nucl. Magn. Reson. Spectrosc.* **2017,** *102–103*, 120–195.
12. Dubroca, T.; Smith, A. N.; Pike, K. J.; Froud, S.; Wylde, R.; Trociewitz, B.; McKay, J.; Mentink-Vigier, F.; van Tol, J.; Wi, S.; Brey, W.; Long, J. R.; Frydman, L.; Hill, S., A quasi-optical and corrugated waveguide microwave transmission system for simultaneous dynamic nuclear polarization NMR on two separate 14.1 T spectrometers. *J. Magn. Res.* **2018,** *289*, 35–44.
13. Bolger, A. M.; Lohse, M.; Usadel, B., Trimmomatic: a flexible trimmer for Illumina sequence data. *Bioinformatics* **2014,** *30* (15), 2114–2120.
14. Peng, Y.; Leung, H. C.; Yiu, S. M.; Lv, M. J.; Zhu, X. G.; Chin, F. Y., IDBA-tran: a more robust *de novo* de Bruijn graph assembler for transcriptomes with uneven expression levels. *Bioinformatics* **2013,** *29* (13), i326–334.
15. Seppey, M.; Manni, M.; Zdobnov, E. M., BUSCO: assessing genome assembly and annotation completeness. *Methods Mol. Biol.* **2019,** *1962*, 227–245.
16. Shekhawat, L. K.; Tiwari, A.; Yamamoto, S.; Rathore, A. S., An accelerated approach for mechanistic model based prediction of linear gradient elution ion-exchange chromatography of proteins. *J. Chromatogr. A.* **2022,** *1680*, 463423.
17. Breil, C.; Abert Vian, M.; Zemb, T.; Kunz, W.; Chemat, F., "Bligh and Dyer" and Folch methods for solid-liquid-liquid extraction of lipids from microorganisms. comprehension of solvatation mechanisms and towards substitution with alternative solvents. *Int. J. Mol. Sci.* **2017,** *18* (4).
18. Arnold, A. A.; Genard, B.; Zito, F.; Tremblay, R.; Warschawski, D. E.; Marcotte, I., Identification of lipid and saccharide constituents of whole microalgal cells by ^{13}C solid-state NMR. *Biochim. Biophys. Acta Biomembr.* **2015,** *1848* (1, Part B), 369–377.
19. Baer, A.; Hoffmann, I.; Mahmoudi, N.; Poulhazan, A.; Harrington, M. J.; Mayer, G.; Schmidt, S.; Schneck, E., The internal structure of the velvet worm projectile slime: A small-angle scattering study. *Small* **2022**, 2300516.
20. Benkendorff, K.; Beardmore, K.; Gooley, A. A.; Packer, N. H.; Tait, N. N., Characterisation of the slime gland secretion from the peripatus, *Euperipatoides kanangrensis* (Onychophora: Peripatopsidae). *Comp. Biochem. Physiol. B, Biochem. Mol. Biol.* **1999,** *124* (4), 457–465.
21. Lu, Y.; Sharma, B.; Soon, W. L.; Shi, X.; Zhao, T.; Lim, Y. T.; Sobota, R. M.; Hoon, S.; Pilloni, G.; Usadi, A.; Pervushin, K.; Miserez, A., Complete sequences of the velvet worm slime proteins reveal that slime formation is enabled by disulfide bonds and intrinsically disordered regions. *Adv. Sci.* **2022,** *9* (18), e2201444.
22. Poulhazan, A.; Dickwella Widanage, M. C.; Muszyński, A.; Arnold, A. A.; Warschawski, D. E.; Azadi, P.; Marcotte, I.; Wang, T., Identification and quantification of glycans in whole cells: architecture of microalgal polysaccharides described by solid-state nuclear magnetic resonance. *J. Am. Chem. Soc.* **2021,** *143* (46), 19374–19388.
23. Sanchez-Ruiz, J. M.; Martinez-Carrion, M., A Fourier-transform infrared spectroscopic study of the phosphoserine residues in hen egg phosvitin and ovalbumin. *Biochemistry* **1988,** *27* (9), 3338–3342.
24. Barja, B. C.; Herszage, J.; dos Santos Afonso, M., Iron(III)-phosphonate complexes. *Polyhedron* **2001,** *20* (15), 1821–1830.
25. Hohwy, M.; Rienstra, C. M.; Jaroniec, C. P.; Griffin, R. G., Fivefold symmetric homonuclear dipolar recoupling in rotating solids: Application to double quantum spectroscopy. *J. Chem. Phys.* **1999,** *110* (16), 7983–7992.
26. Steiner, S.; Conti, S. F.; Lester, R. L. Occurrence of phosphonosphingolipids in *Bdellovibrio bacteriovoru* strain UKi2. *J. Bacteriol.* **1973**, *116* (3), 1199–1211.
27. Acker, M.; Hogle, S. L.; Berube, P. M.; Hackl, T.; Stepanauskas, R.; Chisholm, S. W.; Repeta, D. J. Phosphonate production by marine microbes: exploring new sources and potential function. *bioRxiv* **2020**, 2020.2011.2004.368217.
28. Jayasimhulu, K.; Hunt, S. M.; Kaneshiro, E. S.; Watanabe, Y.; Giner, J.-L. Detection and identification of *Bacteriovorax stolpii* UKi2 sphingophosphonolipid molecular species. *J. Am. Soc. Mass Spectrom.* **2007**, *18* (3), 394–403.
29. Watanabe, Y.; Nakajima, M.; Hoshino, T.; Jayasimhulu, K.; Brooks, E. E.; Kaneshiro, E. S. A novel sphingophosphonolipid head group 1-hydroxy-2-aminoethyl phosphonate in *Bdellovibrio stolpii*. *Lipids* **2001**, *36* (5), 513–519.
30. Yu, X.; Doroghazi, J. R.; Janga, S. C.; Zhang, J. K.; Circello, B.; Griffin, B. M.; Labeda, D. P.; Metcalf, W. W. Diversity and abundance of phosphonate biosynthetic genes in nature. *Proc. Nat. Acad. Sci.* **2013**, *110* (51), 20759–20764.

31. Serrano, A. A.; Schenkman, S.; Yoshida, N.; Mehlert, A.; Richardson, J. M.; Ferguson, M. A. J. The lipid structure of the glycosylphosphatidylinositol-anchored mucin-like sialic acid acceptors of *Trypanosoma cruzi* changes during parasite differentiation from epimastigotes to infective metacyclic trypomastigote forms. *J. Biol. Chem.* **1995**, *270* (45), 27244–27253.
32. Ferguson, M. A.; Allen, A. K.; Snary, D. The detection of phosphonolipids in the protozoan T*rypanosoma cruzi*. *Biochem. J.* **1982**, *207* (1), 171–174.
33. Yu, X.; Doroghazi, J. R.; Janga, S. C.; Zhang, J. K.; Circello, B.; Griffin, B. M.; Labeda, D. P.; Metcalf, W. W. Diversity and abundance of phosphonate biosynthetic genes in nature. *Proceedings of the National Academy of Sciences* **2013**, *110* (51), 20759-20764.
34. Korn, E. D.; Dearborn, D. G.; Fales, H. M.; Sokoloski, E. A. Phosphonoglycan. A major polysaccharide constituent of the amoeba plasma membrane contains 2-aminoethylphosphonic acid and 1-hydroxy-2-aminoethylphosphonic acid. *J. Biol. Chem.* **1973**, *248* (6), 2257–2259.
35. Kennedy, K. E.; Thompson, G. A. Phosphonolipids: Localization in surface membranes of tetrahymena. *Science* **1970**, *168* (3934), 989–991.
36. Wassef, M. K.; Hendrix, J. W. Ceramide aminoethylphosphonate in the fungus Pythium prolatum. *Biochim. Biophys. Acta - Lipids Lipid Metab.* **1977**, *486* (1), 172–178.
37. Kim, H.; Chin, J.; Choi, H.; Baek, K.; Lee, T. G.; Park, S. E.; Wang, W.; Hahn, D.; Yang, I.; Lee, J.; et al. Phosphoiodyns A and B, unique phosphorus-containing iodinated polyacetylenes from a Korean sponge *Placospongia* sp. *Org. Lett.* **2013**, *15* (1), 100–103.
38. Hori, T.; Sugita, M. Sphingolipids in lower animals. *Progress in Lipid Research* **1993**, *32* (1), 25-45.
39. Kariotoglou, D. M.; Mastronicolis, S. K. Sphingophosphonolipid molecular species from edible mollusks and a jellyfish. *Comp. Biochem. Physiol. B, Biochem. Mol. Biol.* **2003**, *136* (1), 27–44.
40. Sugita, M.; Fujii, H.; Dulaney, J. T.; Inagaki, F.; Suzuki, M.; Suzuki, A.; Ohta, S. Structural elucidation of two novel amphoteric glycosphingolipids from the earthworm, Pheretima hilgendorfi. *Biochim. Biophys. Acta - Lipids Lipid Metab.* **1995**, *1259* (3), 220–226.
41. Sugita, M.; Fujii, H.; Dulaney, J. T.; Inagaki, F.; Suzuki, M.; Suzuki, A.; Ohta, S. Structural elucidation of two novel amphoteric glycosphingolipids from the earthworm, Pheretima hilgendorfi. *Biochimica et Biophysica Acta (BBA)/Lipids and Lipid Metabolism* **1995**, *1259* (3), 220-226.
42. Araki, S.; Satake, M.; Ando, S.; Hayashi, A.; Fujii, N. Characterization of a diphosphonopentaosylceramide containing 3-O-methylgalactose from the skin of *Aplysia kurodai* (sea hare). *J. Biol. Chem.* **1986**, *261* (11), 5138–5144.
43. Thébault, M. T.; Kervarec, N.; Pichon, R.; Nonnotte, G.; Le Gal, Y. A ^{31}P nuclear magnetic resonance study of the hydrothermal vent tube worm *Riftia pachyptila*. *C.R. Acad. Sci., Ser. III* **1999**, *322* (7), 537–541.
44. Yamada, S.; Araki, S.; Abe, S.; Kon, K.; Ando, S.; Satake, M. Structural analysis of a novel triphosphonoglycosphingolipid from the egg of the sea hare, *Aplysia kurodai*. *J. Biochem.* **1995**, *117* (4), 794–799.
45. Araki, S.; Abe, S.; Satake, M.; Hayashi, A.; Kon, K.; Ando, S. Novel phosphonoglycosphingolipids containing pyruvylated galactose from the nervous system of *Aplysia kurodai*. *Eur. J. Biochem.* **1991**, *198* (3), 689–695.
46. Satake, M.; Miyamoto, E. A group of glycosphingolipids found in an invertebrate: Their structures and biological significance. *Proc. Jpn. Acad. B: Phys. Biol. Sci.* **2012**, *88* (9), 509–517.
47. Miceli, M. V.; Henderson, T. O.; Myers, T. C. Alkylphosphonic acid distribution in the planorbid snail *Helisoma* sp. *Comp. Biochem. Physiol. B, Comp. Biochem.* **1987**, *88* (2), 603–611.
48. Zhukova, N. V. Lipids and fatty acids of nudibranch mollusks: Potential sources of bioactive compounds. *Mar. Drugs* **2014**, *12* (8), 4578–4592.
49. Matsubara, T.; Hayashi, A. Structural studies on glycolipids of shellfish. V. Gala-6 series glycosphingolipids of the marine snail, *Chlorostoma argyrostoma turbinatum*. *J. Biochem.* **1986**, *99* (5), 1401–1408.
50. Hayashi, A.; Matsubara, T. A new homologue of phosphonoglycosphingolipid, N-methylaminoethylphosphonyltrigalactosylceramide. *Biochim. Biophys. Acta - Lipids Lipid Metab.* **1989**, *1006* (1), 89–96.
51. Matsubara, T.; Morita, M.; Hayashi, A. Determination of the presence of ceramide aminoethylphosphonate and ceramide N-methylaminoethylphosphonate in marine animals by fast atom bombardment mass spectrometry. *Biochim. Biophys. Acta - Lipids Lipid Metab.* **1990**, *1042* (3), 280–286.
52. Matsubara, T.; Hayashi, A. Structural studies on glycolipid of shellfish. III. Novel glycolipids from *Turbo cornutus*. *J. Biochem.* **1981**, *89* (2), 645–650.
53. Saito, H.; Ishikawa, S. Characteristic of lipids and fatty acid compositions of the neon flying squid, *Ommastrephes bartramii*. *J. Oleo. Sci.* **2012**, *61* (10), 547–564.
54. Sinanoglou, V. J.; Miniadis-Meimaroglou, S. Structural analysis of ceramide-amino-ethyl-phosphonate in edible mediterranean cephalopods. *Acta Aliment.* **2004**, *33* (4), 359–370.
55. Itonori, S.; Kamemura, K.; Narushima, K.; Sonku, N.; Itasaka, O.; Hori, T.; Sugita, M. Characterization of a new phosphonocerebroside, N-methyl-2-aminoethylphosphonylglucosylceramide, from the antarctic krill, Euphausia superba. *Biochim. Biophys. Acta - Lipids Lipid Metab.* **1991**, *1081* (3), 321–327.
56. Shimomura, K.; Hanjura, S.; Ki, P. F.; Kishimoto, Y. An unusual glucocerebroside in the crustacean nervous system. *Science* **1983**, *220* (4604), 1392–1393.
57. Baumann, H.; Tzianabos, A. O.; Brisson, J. R.; Kasper, D. L.; Jennings, H. J. Structural elucidation of two capsular polysaccharides from one strain of *Bacteroides fragilis* using high-resolution NMR spectroscopy. *Biochemistry* **1992**, *31* (16), 4081–4089.
58. Coyne, M. J.; Kalka-Moll, W.; Tzianabos, A. O.; Kasper, D. L.; Comstock, L. E. *Bacteroides fragilis* NCTC9343 produces at least three distinct capsular polysaccharides: cloning, characterization, and reassignment of polysaccharide B and C biosynthesis loci. *Infect. Immun.* **2000**, *68* (11), 6176–6181.
59. Tzianabos, A. O.; Pantosti, A.; Baumann, H.; Brisson, J. R.; Jennings, H. J.; Kasper, D. L. The capsular polysaccharide of *Bacteroides fragilis* comprises two ionically linked polysaccharides. *J. Biol. Chem.* **1992**, *267* (25), 18230–18235.
60. Rice, K.; Batul, K.; Whiteside, J.; Kelso, J.; Papinski, M.; Schmidt, E.; Pratasouskaya, A.; Wang, D.; Sullivan, R.; Bartlett, C.; et al. The predominance of nucleotidyl activation in bacterial phosphonate biosynthesis. *Nat Commun* **2019**, *10* (1), 3698.

61. Yu, X.; Price, N. P. J.; Evans, B. S.; Metcalf, W. W. Purification and characterization of phosphonoglycans from *Glycomyces* sp. strain NRRL B-16210 and *Stackebrandtia nassauensis* NRRL B-16338. *J. Bacteriol.* **2014**, *196* (9), 1768–1779.
62. Paschinger, K.; Gutternigg, M.; Rendić, D.; Wilson, I. B. H. The N-glycosylation pattern of *Caenorhabditis elegans*. *Carbohydr. Res.* **2008**, *343* (12), 2041–2049.
63. Paschinger, K.; Gonzalez-Sapienza, G. G.; Wilson, I. B. H. Mass spectrometric analysis of the immunodominant glycan epitope of *Echinococcus granulosus* antigen Ag5. *Int. J. Parasitol.* **2012**, *42* (3), 279–285.
64. Eckmair, B.; Jin, C.; Abed-Navandi, D.; Paschinger, K. Multistep fractionation and mass spectrometry reveal zwitterionic and anionic modifications of the N- and O-glycans of a marine snail. *Mol. Cell Proteomics* **2016**, *15* (2), 573–597.
65. Paschinger, K.; Wilson, I. B. Analysis of zwitterionic and anionic N-linked glycans from invertebrates and protists by mass spectrometry. *Glycoconj J* **2016**, *33* (3), 273-283.
66. Previato, J. O.; Gorin, P. A.; Mazurek, M.; Xavier, M. T.; Fournet, B.; Wieruszek, J. M.; Mendonça-Previato, L. Primary structure of the oligosaccharide chain of lipopeptidophosphoglycan of epimastigote forms of *Trypanosoma cruzi*. *J. Biol. Chem.* **1990**, *265* (5), 2518–2526.
67. Previato, J. O.; Jones, C.; Xavier, M. T.; Wait, R.; Travassos, L. R.; Parodi, A. J.; Mendonça-Previato, L. Structural characterization of the major glycosylphosphatidylinositol membrane-anchored glycoprotein from Epimastigote forms of *Trypanosoma cruzi* Y-strain. *J. Biol. Chem.* **1995**, *270* (13), 7241–7250.
68. de Lederkremer, R. M.; Lima, C.; Ramirez, M. I.; Ferguson, M. A.; Homans, S. W.; Thomas-Oates, J. Complete structure of the glycan of lipopeptidophosphoglycan from *Trypanosoma cruzi* Epimastigotes. *J. Biol. Chem.* **1991**, *266* (35), 23670–23675.
69. Hilderbrand, R. L. *The role of phosphonates in living systems*; 1983. DOI: 10.1201/9781351076470.
70. Quin, L. D. The Presence of compounds with a carbon-phosphorus bond in some marine invertebrates. *Biochemistry* **1965**, *4* (2), 324–330.
71. Urai, M.; Nakamura, T.; Uzawa, J.; Baba, T.; Taniguchi, K.; Seki, H.; Ushida, K. Structural analysis of O-glycans of mucin from jellyfish (*Aurelia aurita*) containing 2-aminoethylphosphonate. *Carbohydr. Res.* **2009**, *344* (16), 2182–2187.
72. Horsman, G. P.; Zechel, D. L. Phosphonate Biochemistry. *Chem Rev* **2017**, *117* (8), 5704-5783.
73. Quin, L. D.; Quin, G. S. Screening for carbon-bound phosphorus in marine animals by high-resolution ^{31}P-NMR spectroscopy: coastal and hydrothermal vent invertebrates. *Comp. Biochem. Physiol. B, Biochem. Mol. Biol.* **2001**, *128* (1), 173–185.
74. Hård, K.; Van Doorn, J. M.; Thomas-Oates, J. E.; Kamerling, J. P.; Van der Horst, D. J. Structure of the asn-linked oligosaccharides of apolipophorin III from the insect *Locusta migratoria*. Carbohydrate-linked 2-aminoethylphosphonate as a constituent of a glycoprotein. *Biochemistry* **1993**, *32* (3), 766–775.
75. Metcalf, W. W.; Griffin, B. M.; Cicchillo, R. M.; Gao, J.; Janga, S. C.; Cooke, H. A.; Circello, B. T.; Evans, B. S.; Martens-Habbena, W.; Stahl, D. A.; et al. Synthesis of methylphosphonic acid by marine microbes: a source for methane in the aerobic ocean. *Science* **2012**, *337* (6098), 1104–1107.
76. Shiraishi, T.; Kuzuyama, T. Biosynthetic pathways and enzymes involved in the production of phosphonic acid natural products. *Biosci. Biotechnol. Biochem.* **2021**, *85* (1), 42–52.
77. Nakashita, H.; Shimazu, A.; Hidaka, T.; Seto, H. Purification and characterization of phosphoenolpyruvate phosphomutase from *Pseudomonas gladioli* B-1. *J. Bacteriol.* **1992**, *174* (21), 6857–6861.
78. Hidaka, T.; Iwakura, H.; Imai, S.; Seto, H. Studies on the biosynthesis of fosfomycin. 3. Detection of phosphoenol-pyruvate phosphomutase activity in a fosfomycin high-producing strain of *Streptomyces wedmorensis* and characterization of its blocked mutant NP-7. *J. Antibiot. (Tokyo)* **1992**, *45* (6), 1008–1010.
79. Hara, O.; Murakami, T.; Imai, S.; Anzai, H.; Itoh, R.; Kumada, Y.; Takano, E.; Satoh, E.; Satoh, A.; Nagaoka, K.; et al. The bialaphos biosynthetic genes of *Streptomyces viridochromogenes*: Cloning, heterospecific expression, and comparison with the genes of *Streptomyces hygroscopicus*. *J. Gen. Microbiol.* **1991**, *137* (2), 351–359.
80. Cioni, J. P.; Doroghazi, J. R.; Ju, K. S.; Yu, X.; Evans, B. S.; Lee, J.; Metcalf, W. W. Cyanohydrin phosphonate natural product from *Streptomyces regensis*. *J. Nat. Prod.* **2014**, *77* (2), 243–249.
81. Ntai, I.; Manier, M. L.; Hachey, D. L.; Bachmann, B. O. Biosynthetic origins of C-P bond containing tripeptide K-26. *Org. Lett.* **2005**, *7* (13), 2763–2765.
82. Zhou, C.; Luo, X.; Chen, N.; Zhang, L.; Gao, J. C-P natural products as next-generation herbicides: chemistry and biology of glufosinate. *J. Agric. Food. Chem.* **2020**, *68* (11), 3344–3353.
83. Horiguchi, M.; Kandatstu, M. Isolation of 2-Aminoethane phosphonic acid from rumen protozoa. *Nature* **1959**, *184* (4690), 901–902.
84. Kleps, R. A.; Myers, T. C.; Lipcius, R. N.; Henderson, T. O. A sex-specific metabolite identified in a marine invertebrate utilizing phosphorus-31 nuclear magnetic resonance. *PLOS ONE* **2007**, *2* (8), e780.
85. Alhadeff, J. A.; Daves, G. D., Jr. Occurrence of 2-aminoethylphosphonic acid in human brain. *Biochemistry* **1970**, *9* (25), 4866–4869.
86. Castronovo, F. P.; Kopiwoda, S.; Peterson, M. 2-aminoethylphosphonic acid: biodistribution of a naturally occurring phosphonate after labelling with technetium-99m. *Nucl. Med. Commun.* **1996**, *17* (10), 902–906.
87. Harrison, J.; Studholme, D. J. Draft genome sequence of *Xanthomonas axonopodis pathovar vasculorum* NCPPB 900. *FEMS Microbiol. Lett.* **2014**, *360* (2), 113–116.
88. Arroyo-Herrera, I.; Rojas-Rojas, F. U.; Lozano-Cervantes, K. D.; Larios-Serrato, V.; Vásquez-Murrieta, M. S.; Whtiman, W. B.; Ibarra, J. A.; Estrada-de Los Santos, P. Draft genome of five *Cupriavidus plantarum* strains: agave, maize and sorghum plant-associated bacteria with resistance to metals. *3 Biotech* **2020**, *10* (6), 242.
89. Deslauriers, R.; Byrd, R. A.; Jarrell, H. C.; Smith, I. C. P. ^{31}P NMR Studies of Vegetative and Encysted Cells of *Acanthamoeba castellanii*. *Eur. J. Biochem.* **1980**, *111* (2), 369–375.
90. Maciejczyk, E.; Wieczorek, D.; Zwyrzykowska-Wodzińska, A.; Halama, M.; Jasicka-Misiak, I.; Kafarski, P. Phosphorus profile of Basidiomycetes. *Phosphorus Sulfur Silicon Relat. Elem.* **2015**, *190*, 763–768.
91. Koukol, O.; Novák, F.; Hrabal, R. Composition of the organic phosphorus fraction in basidiocarps of saprotrophic and mycorrhizal fungi. *Soil Biol. Biochem.* **2008**, *40* (9), 2464–2467.
92. Shelburne, F. A.; Quin, L. D. Isolation of 2-(methylamino)ethylphosphonic acid from the proteinaceous residue of a sea anemone. *Biochim. Biophys. Acta - Gen. Subj.* **1967**, *148* (2), 595–597.

93. Chichibu, S.; Chiba, A. Changes of ^{31}P metabolism during mucus secretion in the slug (*Incilaria bilineata*). *Comp. Biochem. Physiol. C: Comp. Pharmacol.* **1993**, *105* (2), 179–183.
94. Robitaille, P. M.; Kurtz, D. M., Jr. ^{31}P NMR probes of sipunculan erythrocytes containing the O_2-carrying protein hemerythrin. *Biochemistry* **1988**, *27* (12), 4458–4465.
95. Bartlett, C.; Bansal, S.; Burnett, A.; Suits, M. D.; Schaefer, J.; Cegelski, L.; Horsman, G. P.; Weadge, J. T. Whole-cell detection of C-P bonds in bacteria. *Biochemistry* **2017**, *56* (44), 5870–5873.
